# The involvement of TGF-β1 /FAK/α-SMA pathway in the antifibrotic impact of rice bran oil on thioacetamide-induced liver fibrosis in rats

**DOI:** 10.1371/journal.pone.0260130

**Published:** 2021-12-29

**Authors:** Rehab F. Abdel-Rahman, Hany M. Fayed, Gihan F. Asaad, Hanan A. Ogaly, Alyaa F. Hessin, Abeer A. A. Salama, Sahar S. Abd El-Rahman, Mahmoud S. Arbid, Marawan Abd Elbaset Mohamed

**Affiliations:** 1 Department of Pharmacology, Medical Research and Clinical Studies Institute, National Research Centre, Giza, Egypt; 2 Department of Chemistry, College of Science, King Khalid University, Abha, Kingdom of Saudi Arabia; 3 Department of Biochemistry, College of Veterinary Medicine, Cairo University, Giza, Egypt; 4 Department of Pathology, Faculty of Veterinary Medicine, Cairo University, Giza, Egypt; Zagazig University, EGYPT

## Abstract

The objective of the current study is to investigate the effect of rice bran oil (RBO) on hepatic fibrosis as a characteristic response to persistent liver injuries. Rats were randomly allocated into five groups: the negative control group, thioacetamide (TAA) group (thioacetamide 100 mg/kg thrice weekly for two successive weeks, ip), RBO 0.2 and 0.4 groups (RBO 0.2mL and 0.4 mL/rat/day, *po*) and standard group (silymarin 100 mg/kg/day, *po*) for two weeks after TAA injection. Blood and liver tissue samples were collected for biochemical, molecular, and histological analyses. Liver functions, oxidative stress, inflammation, liver fibrosis markers were assessed. The obtained results showed that RBO reduced TAA-induced liver fibrosis and suppressed the extracellular matrix formation. Compared to the positive control group, RBO dramatically reduced total bilirubin, AST, and ALT blood levels. Furthermore, RBO reduced MDA and increased GSH contents in the liver. Simultaneously RBO downregulated the NF-κβ signaling pathway, which in turn inhibited the expression of some inflammatory mediators, including Cox-2, IL-1β, and TNF-α. RBO attenuated liver fibrosis by suppressing the biological effects of TGF-β1, α-SMA, collagen I, hydroxyproline, CTGF, and focal adhesion kinase (FAK). RBO reduced liver fibrosis by inhibiting hepatic stellate cell activation and modulating the interplay among the TGF-β1 and FAK signal transduction. The greater dosage of 0.4 mL/kg has a more substantial impact. Hence, this investigation presents RBO as a promising antifibrotic agent in the TAA model through inhibition of TGF-β1 /FAK/α-SMA.

## Introduction

Liver fibrosis is a dynamic process of repetitive liver injury, which eventually leads to cirrhosis and organ failure [1]. It is a considerable health problem that is associated with significant morbidity and mortality worldwide. The principal causative factors of liver fibrosis in developing countries are hepatitis C virus and/or parasitic infections, while in developed countries, the frequent causes are hepatitis B virus and excessive alcohol consumption [2]. Numerous stimuli are known to cause chronic liver inflammation and hepatic fibrosis, including but not limited to autoimmune disorders, metabolic disorders, drug or toxins-induced diseases, chronic cholestatic diseases, and genetic diseases [3]. Despite various etiologies, fibrosis represents a hallmark of all chronic liver diseases; if left, it progresses to cirrhosis, hepatocellular carcinoma, and eventually death [4]. Progressive liver fibrosis represents significant risk factor for developing liver-related complications and mortality [5].

Hepatic stellate cells (HSCs) exist in the space of Disse among hepatocytes and sinusoidal endothelial cells in the liver. One of its distinguishing characteristics is that they retain vitamin A lipids in their cytosol [6]. Upon repeated liver injury, quiescent HSCs become activated and differentiated into myofibroblast‐like cells; they lose their stored vitamin A and lipids; express α-smooth muscle actin (α-SMA), and produce large amounts of extracellular matrix (ECM) proteins [7]. Many cellular and molecular mechanisms implicated in HSC activation and consequent fibrogenesis, including cytokines and reactive oxygen species, thus offers probable therapeutic targets [2, 8]. The focal adhesion kinase (FAK) signaling pathway is among the multiple signaling pathways that participate in the fibrogenic process is the focal adhesion kinase (FAK) signaling pathway.

Focal adhesion kinase is a cytoplasmic tyrosine kinase that plays a pivotal role in liver fibrosis via activation and differentiation of HSC, stimulation of myofibroblast proliferation, and resistance to apoptosis. Additionally, inhibition of FAK signaling by drugs may be a potential target for preventing liver fibrosis [9]. FAK activation is vital for the expression of α-SMA and pro-fibrotic collagens from hepatic stellate cells -[10]. In the fibrotic liver, the FAK mRNA levels are higher than in the healthy liver [11]. FAK is a potential mediator of fibrosis via fibroblast attachment to the extracellular matrix (ECM), and according to previous studies, it downregulates signaling that is implicated in two main fibrosis mechanisms. Phosphatidylinositol 3-kinase/protein kinase B (PI3K/AKT) signaling pathway is known as a mammalian target of rapamycin/S6 kinase (mTOR/S6K) complex. At the same time, the extracellular signal is controlled by ERK1/2, which is one of mitogen-activated protein kinase (MAPK) signal transduction. These pathways can result in α-SMA-positive myofibroblast diversity as well as collagen formation of various types, indicating the progression of liver fibrosis [12]. As a result, pharmacological suppression of FAK might be a viable treatment option for liver fibrosis. In this case, natural bioactive components from plant materials, particularly plant products, may be helpful. Meanwhile, RBO, which is derived from such plant sources, is a promising candidate [13].

Rice bran oil is becoming more widely used around the globe. It is a rice processing industry by-product that is removed from the white rice during the curing process [14]. Oryzanols, tocotrienols, tricin, phytosterols, policosanols, squalene, tocopherols, and ferulic acid are only some of the bioactive components that are found in RBO [15, 16]. Various health advantages have been claimed for RBO, particularly cholesterol-lowering [17], anti-inflammatory [18], and antioxidant activities [19]. Additionally, RBO is an edible oil available in the local market and is rich in many commercially and nutritionally essential phytochemicals such as oryzanol, lecithin, and tocotrienols. Many of these phytochemicals are removed as waste by-products of RBO during the refining process. One of such components is γ-oryzanol which is a mixture of ferulic acid esters of sterol and triterpene alcohols [20]. RBO contains oryzanol at a level of 1 to 2%, where it acts as a natural antioxidant that can reduce blood cholesterol levels and lowering the risk of coronary heart disease [21]. The chemo-preventive activity of rice bran-derived components has been related to bioactive phytochemicals such as ferulic acid, tocotrienols/tocopherols, tricin, β-sitosterol, γ-oryzanol, and phytic acid. The underlying mechanisms may be mediated through their ability to inhibit cell proliferation, induce apoptosis, and alter cell cycle progression in cancer cells. Moreover, rice bran bioactive components could protect against tissue damage through the radical scavenging activity and blocking the chronic inflammatory responses [22].

RBO is a balanced source of saturated fatty acids (SFA: 20% palmitic acid), monounsaturated (MUFA: 42% oleic acid), and polyunsaturated fatty acid (PUFA: 32% linoleic acid) with an average ratio of 0.6:1.1:1.0, respectively. Moreover, it is of better oxidative stability than other cooking oils, with a high smoke point of 232°C and an ignition point of 350°C, making it suitable for the high-temperature cooking process [23]

Interestingly, rice bran and its oil are rich in tricin, a natural flavonoid with anti-inflammatory properties that show a variety of biological actions by inhibiting NF- κβ signaling and therefore reducing the release of pro-inflammatory cytokines [24, 25]. Moreover, tricin exhibited anticancer effects by suppressing the FAK protein levels and its downstream signals [26].

Based on the literature supporting the anti-inflammatory and antioxidant properties of rice bran oil and its constituents, the current study aimed at scouting the potential antifibrotic effects of RBO against TAA-induced liver fibrosis in rats and investigating whether the selected oil could abate this dysregulation and suppress the inflammatory mediators and oxidative stress. Moreover, to date, no data is available to link the regulation of the TGF-β1/FAK/α-SMA pathway to the antifibrotic value of rice bran oil against TAA-induced liver fibrosis. Therefore, the current study aimed to investigate the potential antifibrotic effects of RBO against TAA-induced liver fibrosis in rats and the involvement of the TGF-β1/FAK/α-SMA pathway to TAA-induced liver fibrosis.

## Materials and methods

### Animals

Thirty adult male Wistar rats, five months of age, weighing 150–200 g, were purchased from the “Animal House Colony at the National Research Centre (NRC, Egypt)”. Rats were kept at room temperature (25°C) with a 12 h light and 12 h dark cycle. The animals were treated according to the national and international ethics guidelines. All experimental methods were carried out in compliance with the ethical criteria authorized by the “NRC’s Committee on Animal Care and Use’s ethics committees” (approval number: MREC-19-214).

### Chemicals

Thioacetamide (TAA) was purchased from “Sigma-Aldrich, USA”. Silymarin was obtained from “MEPACO, Egypt, and rice bran oil (RBO) was procured from “KING®, Thai Edible Oil Co., Ltd., Bangkok, Thailand”. All other chemicals used within the study were from the highest analytical grade accessible.

### GC-MS chromatogram analysis

The GC/MS analysis was performed using a Thermo Scientific, Trace GC Ultra/ISQ Single Quadrupole MS, TG-5MS fused silica capillary column (30m, 0.251mm, 0.1mm film thickness). For GC/MS detection, an electron ionization system with ionization energy of 70 eV was used. Helium gas was used as the carrier gas at a constant flow rate of 1mL/min. The injector and MS transfer line temperature was set at 280°C. The oven temperature was programmed at an initial temperature of 50°C (hold 2 min) to150°C at an increasing rate of 7°C/min. Then to 270 at an increasing rate of 5°C/min (hold 2 min) then to 310 as a final temperature at an increasing rate of 3.5°C/min (hold 10 min).

The quantification of all the identified components was investigated using a percent relative peak area. Tentative identification of the compounds was performed based on comparing their relative retention time and mass spectra with those of the NIST, WILLY library data of the GC/MS system.

### Experimental design

After a one-week of acclimatization, rats were divided into five groups, each with six animals, according to the following scheme: **Group 1:** the negative control group; rats were injected intraperitoneally (*ip*) with saline three times per week for two successive weeks, **Group 2:** the positive control (TAA) group; rats were *ip* injected with TAA (100 mg/kg) three times per week for two successive weeks to provoke liver fibrosis [27] () with some modification based on our preliminary studies. **Group 3 and 4:** treatment groups; rats received orally RBO (0.2 and 0.4 mL/kg, orally) [28, 29] daily for 2 weeks after 2 weeks of TAA injection. **Group 5:** reference group; rats received silymarin (100 mg/kg, orally) [30], daily for 2 weeks after 2 weeks of TAA injection.

### Preparation of blood and tissue samples

At the end of the experiment, blood samples were collected from the retro-orbital venous plexus of each rat under mild ketamine anesthesia. Serum samples were extracted from the blood samples and stored at -20°C for subsequent biochemical analysis. Ketamine anesthetized rats were euthanized by cervical dislocation directly after blood sample, and livers were quickly removed, cleaned in ice-cold saline, blotted dry, and weighed. The left lobe of each rat’s liver was dissected out and put in 10% buffered neutral formalin for histopathological and immunohistochemical examinations, while another weighted portion was preserved frozen at -80°C for subsequent molecular and biochemical studies.

To create a 20 percent w/v homogenate, the later weighed component of each hepatic tissue was homogenized with ice-cooled saline using a homogenizer “Medical equipment, MPW-120, Poland”. To eliminate cell debris, the homogenate was centrifuged at 4000 rpm for 5 minutes at 4°C in a cooling centrifuge “Laborzentrifugen, 2k15, Sigma, Germany”. Aliquots were then stored at -80°C for biological examination.

### Determination of liver function

Serum activities of aspartate aminotransferase (AST) (Catalog # AS 10 61 (45), Biodiagnostic, Egypt) and alanine aminotransferase (ALT) (Catalog # AL 10 31 (45), Biodiagnostic, Egypt) were determined colorimetrically using commercial Biodiagnostic® kits, Egypt.

### Determination of oxidative stress biomarkers

Liver contents of reduced glutathione (GSH) Assay Kit (Catalog # GR 25 10, Biodiagnostic, Egypt), Malondialdehyde (MDA) Colorimetric/Fluorometric Assay Kit (Catalog # MD 25 28, Biodiagnostic, Egypt), were determined colorimetrically according to Ellman [31]‎, and Ruiz-Larrea et al. ‎[32], respectively, using standard chemical methods.

### Determination of pro-fibrotic and inflammatory markers

Liver contents of tumor necrosis factor-alpha (TNF-α) (Catalog# SL0889Mo, Sunlong Biotec Co. LTD, Zhejiang, China), nuclear factor-kappa B (NF- κβ), interleukin 1 beta (IL-1β), transforming growth factor β1 (TGF-β1) (Catalog# SL1423Ra, Sunlong Biotec Co. LTD, Zhejiang, China), alpha-smooth muscle actin (α-SMA) (Catalog# SL0988Ra, Sunlong Biotec Co. LTD, Zhejiang, China), collagen I, hydroxyproline, connective tissue growth factor (CTGF) (Catalog# SL0196Ra, Sunlong Biotec Co. LTD, Zhejiang, China) and focal adhesion kinase (FAK) (Catalog# SL0721Hu, Sunlong Biotec Co. LTD, Zhejiang, China) were estimated using ELISA kits according to the manufacturing instructions of Sunlong Biotec Co. LTD, Zhejiang, China.

### Histopathological examination

Tissue specimens of liver from rats of various groups were consistently prepared for paraffin slices after twenty-four hours of fixation in 10% buffered neutral formalin. The specimens were washed with distilled water, dehydrated in repeated dilutions of ethanol, cleared in xylene, and lastly embedded in paraffin. Four to five μm thick sections of paraffin blocks were cut. The tissue slices were mounted on glass slides, deparaffinized, and stained with hematoxylin and eosin (H&E) and Masson’s Trichrome stains (MTC) [33]. A light microscope (Olympus, Germany) was used to examine the obtained slides. Fibrosis was assessed using the Metavir grading method (ranged from F0 = no fibrosis to F4 = cirrhosis) [34]. The MTC stained regions of fibrous tissue were quantified as an area percentage using image analysis software (Image J, 1.46a, NIH, USA).

All histopathological examinations were conducted by an expert investigator who was blinded during sample identification to prevent bias.

### Immunohistochemical studies

PDGF-BB and p-AKT liver content were detected using “avidin-biotin-peroxidase (DAB, Sigma Chemical Co.)” on paraffin slices of the liver of the control and all treatment groups, similar to the technique described by [35]. Tissue sections were incubated with a monoclonal antibody for PDGF-BB and p-AKT (Abcam, Cambridge, MA, USA, ab9704 and ab8805 respectively) at 1:200 and 1:100 dilutions respectively; and reagents required for the avidin-biotin-peroxidase (Vactastain ABC peroxidase kit, Vector Laboratories) method for the detection of the “antigen-antibody complex.” Each marker expression was visualized by the chromogen “3,3 -diaminobenzidine tetrahydrochloride (DAB, Sigma Chemical Co.)”. Quantification of the positive brown area of each marker’s expression was implemented as an optical density in 7 high-power microscopic fields using image analysis software (Image J, 1.46a, NIH, USA).

### Comparative RT-qPCR

Total RNA was extracted from frozen liver samples using TRIzol reagent and RNase-Free DNase Set (Thermo Fisher Scientific, USA) following the instructions provided by the manufacturer [36]. RNA purity and concentration were assessed by spectrophotometry Thermo Scientific, Wilmington, DE, USA. The first-strand cDNA was synthesized by using SuperScript IV VILO cDNA Reverse Transcription System (Invitrogen). To measure the mRNA expression, qPCR was performed in triplicate for each sample using SYBR® Premix Ex Taq TM (Life Technologies). The oligonucleotide primers used for amplification of the target genes (NF- κβ, COX-2, and β-actin) were obtained from Invitrogen **(Table 1)**. The relative mRNA quantification of each gene was normalized to that of β-actin and calculated using the 2−ΔΔCt method.

**Table 1:** List of “oligonucleotide primers” used in qPCR.

Gene		Sequence (5′-3′)	Accession #
**NF-κβ**	F	CTGGCAGCTCTTCTCAAAGC	XM_006233360.3
	R	CCAGGTCATAGAGAGGCTCAA	
**COX-2**	F	AAAGCC TCGTCCAGATGCTA	NM_017232.3
	R	ATGGTGGCTGTCTTGGTAGG	
**β-actin**	F	AT GGTGGGTATGGGTCAG	NM_031144.3
	R	CAATGCCGTGTTCAATGG	

### Statistical analysis

The values are expressed as mean ± standard error of six observations in each group. All groups were subjected to one-way analysis of variance (ANOVA), which was followed by Tukey’s multiple comparisons test to determine the intergroup variability by using Graphpad Prism® software, version 8 (Inc., San Diego, USA). When the difference was *p*≤0.05, it was judged significant.

## Results

### GC-MS chromatogram of RBO analysis

**Fig 1:**
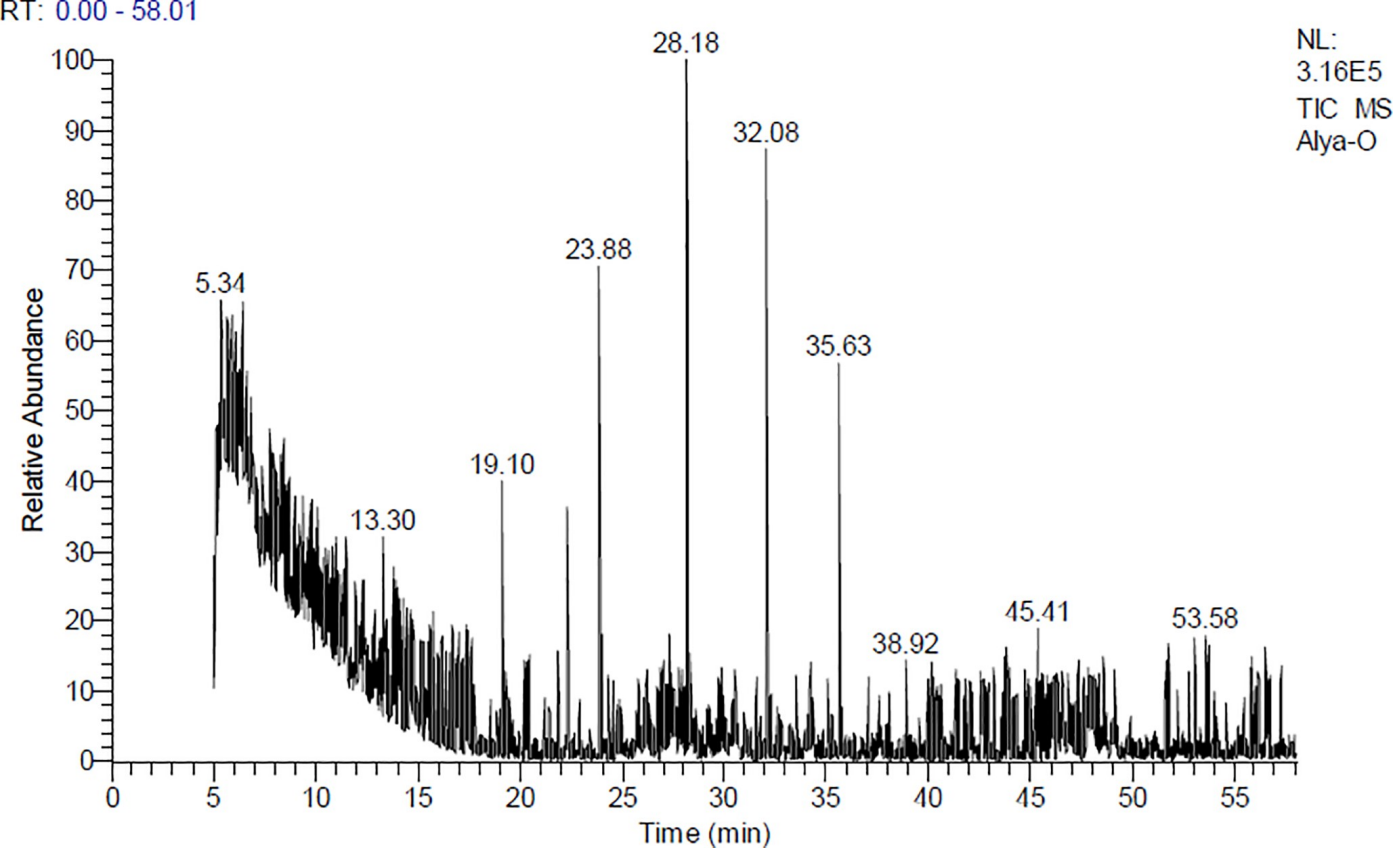
GC-MS chromatogram of RBO analysis.

GC/MS analysis of rice bran oil consists of 10 compounds. The total peak areas of the detected compounds are 100%, the probabilities of the structures of the detected compounds are listed in **(Table 2)** and **(Fig 1)**: The major compounds are Cyclooctane, butyl- (13.17%), 9-Tricosene, (Z)- (CAS) (18.98%), 1-Octadecanol (CAS) (15.43%) and 1-Heptadecene (CAS) (9.30%) for which represented (56.88%), of the total peak areas. The identification was accomplished using computer search user-generated reference libraries, incorporating mass spectra. Peaks were examined by single-ion chromatographic reconstruction to confirm their homogeneity. In some cases, when identical spectra have not been found, only the structural type of the corresponding component was proposed based on its mass spectral fragmentation. Reference compounds were co-chromatographed, when possible, to confirm GC retention times.

**Table 2:** Chemical characterization of rice bran oil via GC-MS analysis.

Peak No.	R_t_ (min.)	MW	MF	Area%	Probabilities of the detected compounds
**1**	5.17	129	CHCl2NO2	6.51	Methane, dichloronitro-
**2**	5.34	676	C44H27DN4Zn	7.54	(5,10,15,20-tetraphenyl[2-(2)H1]prophyrinato)zinx(II)
**3**	5.43	650	C40H58Si4	8.92	1,4-Di-hept-1-ynyl-2,3,5, 6-tetrakis(trimethylsilylet hynyl)benzene
**4**	8.70	648	C30H52O6Si5	6.02	1-[2,4,6-tris(trimethylsiloxy)phenyl]-3-[3,4-di(trimet hylsiloxy)phenyl]-2-propen-1-one
**5**	19.11	140	C10H20	6.49	4-Nonene, 3-methyl-, (Z)
**6**	22.32	206	C14H22O	7.64	Phenol, 2,4-bis(1,1-dimethylethyl)
**7**	23.88	168	C12H24	13.17	Cyclooctane, butyl-
**8**	28.17	322	C23H46	18.98	9-Tricosene, (Z)- (CAS)
**9**	32.08	270	C18H38O	15.43	1-Octadecanol (CAS)
**10**	35.64	238	C17H34	9.30	1-Heptadecene (CAS)

R_t_: Retention time MF: Molecular formula MW: Molecular weight.

### Liver transaminases

**Fig 2:**
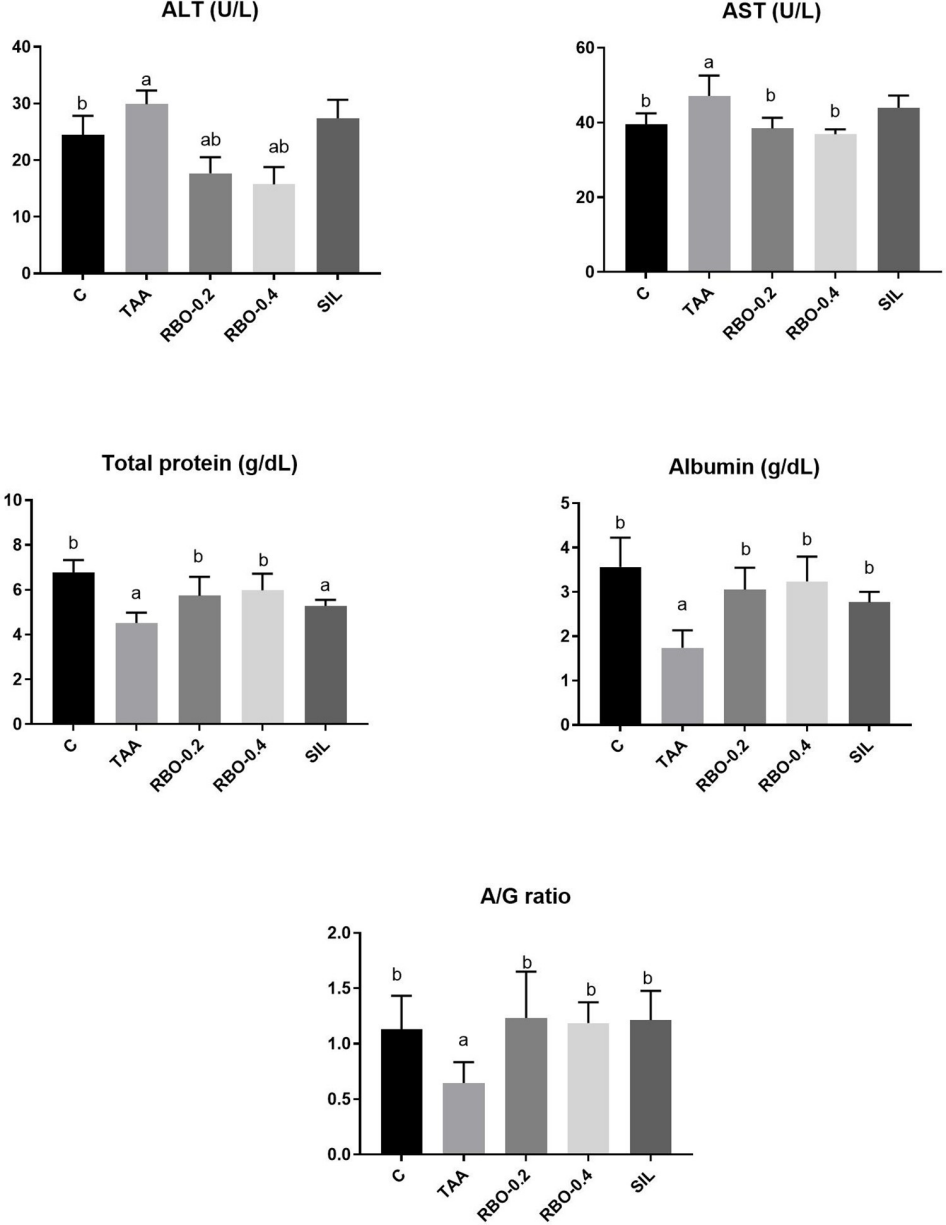
Effect of RBO on serum levels of ALT, AST, total protein, albumin and A/G ratio in TAA-induced liver fibrosis in rats. Control rats, treated with saline; TAA rats, treated with thioacetamide (100 mg/kg; three times per week for 2 weeks, *ip*); RBO rats, treated with TAA and RBO (0.2 and 0.4 mL/rat; daily for 2 weeks, *po*) and silymarin (100 mg/kg, *po*). All data are presented as Mean± SEM, (n = 6). ^a^ *P*≤0.05 was assumed to denote statistical significance compared to the negative control, ^b^ *P*≤0.05 was assumed to denote statistical significance compared to TAA group. TAA, Thioacetamide; RBO, Rice bran oil; A/G, Albumin/Globulin.

Intraperitoneal injection of TAA (100 mg/kg) three times weekly for two weeks markedly (*p*≤0.05) increased liver transaminases (ALT; 29.9±0.98 U/L and AST; 47.1±2.22 U/L) in serum, showing a percent of elevation (22.5% and 19.24%, respectively) as compared to the negative control group (ALT; 24.4±1.4 U/L and AST; 39.5±1.21 U/L). Groups treated with RBO (0.2 and 0.4 ml/rat) for two weeks after inducing liver fibrosis showed a substantial (*P*≤0.05) reduction of liver enzymes (ALT; 17.7±1.15 and 15.7±1.26 U/L and AST; 38.4±1.167 and 36.9±0.53 U/L) showing the percent of reduction of 40.8% and 47.5% for ALT, 18.47% and 21.65 for AST, respectively. Notably, RBO improves liver enzymes level in a dose corresponding manner. This effect was better than that of the reference drug, silymarin, which showed no significant change (*p*≤0.05) (ALT; 27.4±1.33 U/L and AST; 44.0±1.33 U/L) as compared to the positive control group. Data is represented in **(Fig 2)**.

### Serum levels of total protein, albumin, and A/G ratio

Intraperitoneal injection of TAA (100 mg/kg) three times weekly for two weeks noticeably (*p*≤0.05) decreased in serum total protein (4.5±0.19 g/dL), albumin concentration (1.7±0.16 g/dL) showing a percent of reduction (33.82% and 52.78%) respectively as compared to the negative control group (6.8±0.23, 3.6±0.27 g/dL), as well as a reduction of the A/G ratio (0.65±0.08) as compared to the negative control group (1.13±0.12). Rat groups treated with RBO at both tested dose levels (0.2 and 0.4 mL/rat) for two weeks post-induction of the liver revealed a substantial increase in serum total protein level (1.23±0.23 g/dL and 1.19±0.08, respectively), serum albumin (3.1±0.2 g/dl and 3.2±0.23 g/dL, respectively) as compared to the positive control group. As well, groups treated with RBO (0.2 and 0.4 mL/rat) and silymarin (100 mg/dL) showed normal A/G ratio (1.23±0.17, 1.19±0.08, and 1.21±0.11, respectively), as compared to the negative control group. Data is represented in **(Fig 2)**.

### Effect on oxidative stress indicators in liver tissue

**Fig 3:**
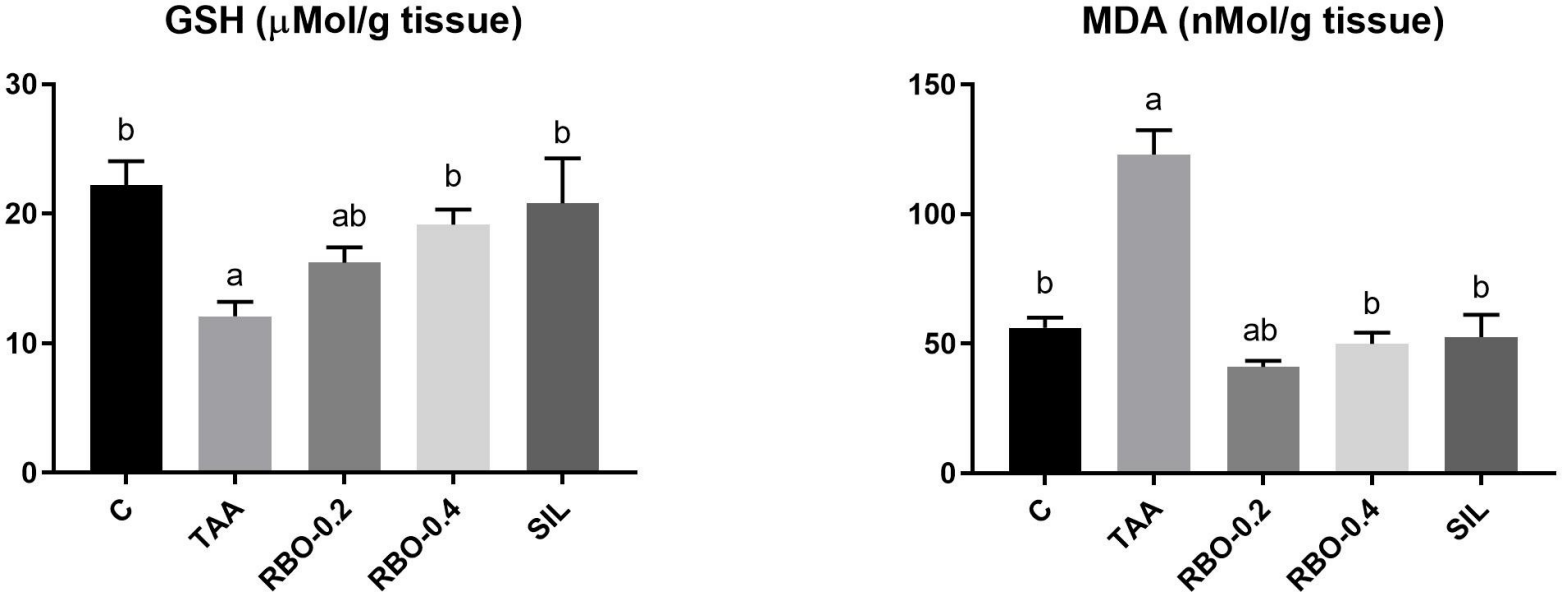
Effect of RBO on tissue levels of GSH and MDA in TAA-induced liver fibrosis in rats. Control rats, treated with saline; TAA rats, treated with thioacetamide (100 mg/kg; three times per week for 2 weeks, *ip*); RBO rats, treated with TAA and RBO (0.2 and 0.4 mL/rat; daily for 2 weeks, *po*) and silymarin (100 mg/kg, *po*). All data are presented as Mean± SEM, (n = 6). ^a^ *P*≤0.05 was assumed to denote statistical significance compared to the negative control, ^b^ *P*≤0.05 was assumed to denote statistical significance compared to TAA group. TAA, Thioacetamide; RBO, Rice bran oil; GSH, Reduced glutathione; MDA, Malondialdehyde.

As shown in **(Fig 3)**, TAA administration resulted in substantial (*p*≤0.05) diminution of hepatic GSH content by 45.49% but was rescued by RBO or silymarin administration. GSH was significantly increased in rat group-administered RBO (0.4 mL/rat) by 58.67%, compared to the TAA-treated group. Data revealed an upsurge in MDA level in the positive control group compared to the negative control group by 119.04%. However, MDA levels were considerably lower in the RBO-treated groups (0.4 mL/rat) by 59.46%, compared to the TAA-treated group.

### Proinflammatory cytokines (TNF-α and IL-1β) and proinflammatory cytokine regulator (NF- κβ)

Intraperitoneal injection of TAA (100 mg/kg) thrice weekly for 2 weeks noticeably (*p*≤0.05) increased TNF-α, IL-1β and NF-κβ (817.5±54.00 ng/mL, 770.0±43.32 pg/mL and 25890.0±1896.66 pg/mL, respectively) in hepatic tissue homogenate as compared to negative control group (TNF-α; 274.3±16.35 ng/mL, IL-1β; 269.4±17.11 pg/mL and NF-κβ; 3626.0±259.61 pg/mL), showing a percent of elevation (198.0%, 185.8% and 85.99%), respectively. Groups treated with RPO at both tested levels (0.2 & 0.4 ml/rat) for two weeks post-induction of liver fibrosis significantly (P≤0.05) decreased TNF-α (422.6±35.71 and 372.7±25.93 ng/L), with percent of reduction (48.31% & 54.41%) respectively, IL-1β (301.9±23.22 and 292.4±11.12 pg/ml) respectively and showing a percent of reduction (60.79% and 62.02%) and NF-κβ (4869.4±490.68 and 4278.8±293.41 pg/mL, respectively) and showing a percent of reduction (81.2% and 83.47%, respectively) as compared to control positive group. Groups treated with RBO at both tested levels (0.2 and 0.4 mL/rat) showed no significant change from the group treated with silymarin in all inflammatory parameters (TNF-α; 335.9±19.48 ng/mL, IL-1β; 375.1±35.51 pg/mL and NF-κβ; 4370.0±221.75 pg/mL) that showed the percent of reduction (TNF-α; 58.92%, IL-1β; 51.28% and NF-κβ; 83.12%). Data are illustrated in **Table 3**.

**Table 3:** Impact on inflammatory mediators (TNF-, IL-1, NF-κβ, collagen I, CTGF and hydroxyproline) in liver tissue.

Group	Liver Homogenate					
	TNF-α (ng/L)	IL-1β (pg/mL)	NF-κβ (pg/mL)	Collagen I (ng/mL)	CTGF (pg/mL)	Hydroxyproline (ng/mL)
**Negative control**	274.3 ^b^ ± 16.35	269.4 ^b^ ± 17.11	3626.0 ^b^ ± 259.61	32.5 ^b^ ± 1.82	1574.0 ^b^ ± 109.08	3098.3 ^b^ ± 148.50
**Positive control (TAA)**	817.5 ^a^ ± 54.00	770.0 ^a^ ± 43.32	25890.0 ^a^ ± 1896.66	64.9 ^a^ ± 4.76	2038.4 ^a^ ± 60.09	4824.4 ^a^ ± 232.66
**RBO (0.2 mL/rat)**	422.6 ^a^^b^ ± 35.71	301.9 ^b^ ± 23.22	4869.4 ^b^ ± 490.68	38.4 ^b^ ± 2.19	1666.6 ^b^ ± 57.80	3707.3 ^b^ ± 265.13
**RBO (0.4 mL/rat)**	372.7 ^b^ ± 25.93	292.4 ^b^ ± 11.12	4278.8 ^b^ ± 293.41	36.4 ^b^ ± 2.99	1564.6 ^b^ ± 55.34	3137.9 ^b^ ± 258.49
**Silymarin (100 mg/kg)**	335.9 ^b^ ± 19.48	375.1 ^b^ ± 35.51	4370.0 ^b^ ± 221.75	36.1 ^b^ ± 2.69	1671.9 ^b^ ± 46.63	3699.4 ^b^ ± 186.91

Values are expressed as Mean ± SEM of six animals in each group.

^a^ Significantly different from the values of the negative control rats at *p*≤0.05.

^b^ Significantly different from the values of the TAA-administered rats at *p*≤0.05.

### Liver fibrosis biomarkers (Hydroxyproline, collagen I, and CTGF)

TAA injection (100 mg/kg, *ip*) thrice weekly for 2 weeks noticeably (*p*≤0.05) increased hydroxyproline, collagen I and CTGF (4824.4±232.66 ng/mL, 64.9±4.76 ng/mL and 2038.4±60.09 pg/mL, respectively) in tissue homogenate, as compared to negative control group (3098.3±148.50 ng/mL, 32.5±1.82 ng/mL and 1574.0±109.08 pg/mL, respectively), showing a percent of elevation (35.78%, 49.92% and 22.78%, respectively). Groups treated with RBO (0.2–0.4 mL/rat) for two weeks post-induction of liver fibrosis showed a significant (*p*≤0.05) reduction of liver hydroxyproline (3707.3±265.13 and 3137.9±258.49 ng/mL), collagen I (38.4±2.19 and 36.4±2.99 ng/mL) and CTGF (1666.6±57.80 and 1564.6±55.34 pg/mL) as compared to control positive group and showing % of reduction in hydroxyproline (23.15% and 34.96%), collagen I (40.83% and 43.91%) and CTGF (18.24% and 23.24%). Groups treated with RBO (0.4 mL/rat) showed no considerable change with the group treated with silymarin hydroxyproline, collagen I, and CTGF (3699.4±186.91 ng/mL, 36.1±2.69 ng/mL, and 1671.9±46.63 pg/mL, respectively) with % of reduction of hydroxyproline, collagen I and CTGF (23.31%, 44.38%, and 44.38%, respectively). Data are illustrated in **Table 3**.

### TGF-β1 /FAK/α-SMA pathway

**Fig 4:**
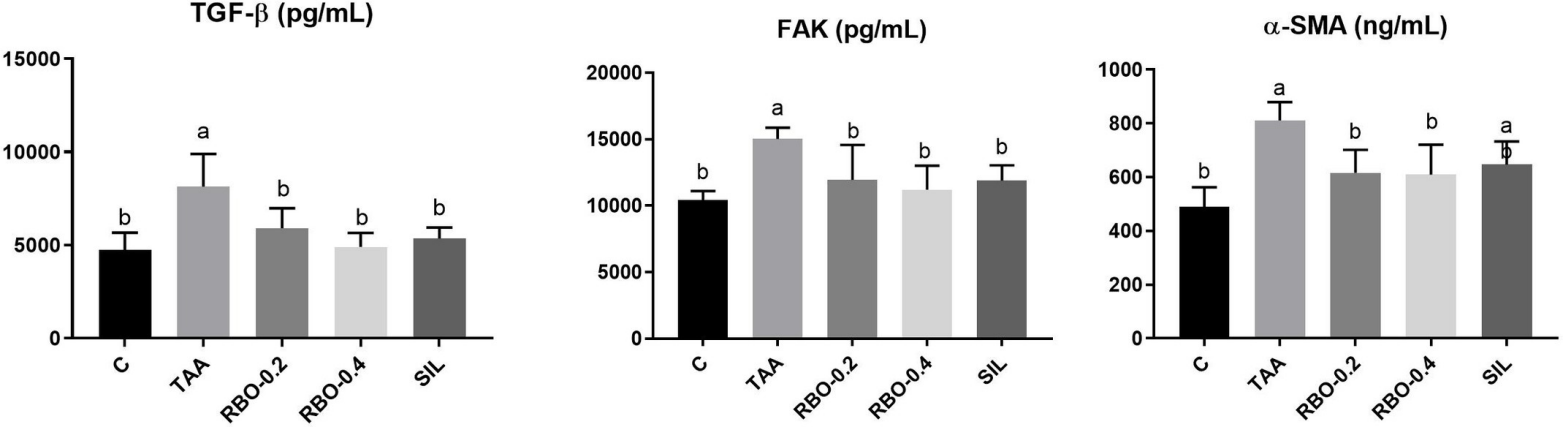
Effect of RBO on TGF-β1, FAK and α-SMA in TAA-induced liver fibrosis in rats. Control rats, treated with saline; TAA rats, treated with thioacetamide (100 mg/kg; three times per week for 2 weeks, *ip*); RBO rats, treated with TAA, RBO (0.2 and 0.4 mL/rat; daily for 2 weeks, *po*) and silymarin (100 mg/kg, *po*). All data are presented as Mean± SEM, (n = 6). ^a^ *P*≤0.05 was assumed to denote statistical significance compared to the negative control, ^b^ *P*≤0.05 was assumed to denote statistical significance compared to TAA group. TAA, Thioacetamide; RBO, Rice bran oil; TGF-β1, Transforming growth factor beta; FAK, Focal adhesion kinase; α-SMA, Alpha-Smooth Muscle Actin.

TAA injection (100 mg/kg, *ip*) thrice weekly for 2 weeks noticeably (*p*≤0.05) increased TGF-β1, FAK and α-SMA (4743.8±376.56 pg/mL, 15022.3±345.60 pg/mL and 811.5±27.91 ng/mL, respectively) in tissue homogenate, as compared to negative control group (4743.8±376.56 pg/mL, 10402.9±286.35 pg/mL and 489.5±30.07 ng/mL, respectively), showing a percent of elevation (41.73%, 30.75% and 39.68%, respectively). Groups treated with RBO (0.2 and 0.4 mL/rat) for two weeks post-induction of liver fibrosis showed a significant (*p*≤0.05) reduction of liver TGF-β1 (5902.0±440.72 and 4905.7±305.98 pg/ml), FAK (11935.1±1079.75 and 11204.8±734.83 pg/mL), and α-SMA (616.4±34.81 and 11204.8±734.83 pg/mL) as compared to control positive group and showing % of reduction in TGF-β (27.5% and 39.74%), FAK (20.55% and 25.41%) and α-SMA (24.04% and 24.86%). Groups treated with RBO (0.4 mL/rat) showed no considerable change with the group treated with silymarin in TGF-β1 and FAK (5351.6±240.28 and 11889.6±471.32 pg/mL, respectively) with % of reduction of TGF-β1 and FAK (34.26% and 20.85%, respectively). Whereas, the group treated with RBO (0.4 mL/rat) showed better results than that exerted by silymarin, which still showed significant elevation (647.2±34.93 ng/mL) compared to the negative control % reduction = 20.24% as compared to the control positive group. Data are illustrated in **(Fig 4)**.

### Histopathological investigation

**Fig 5:**
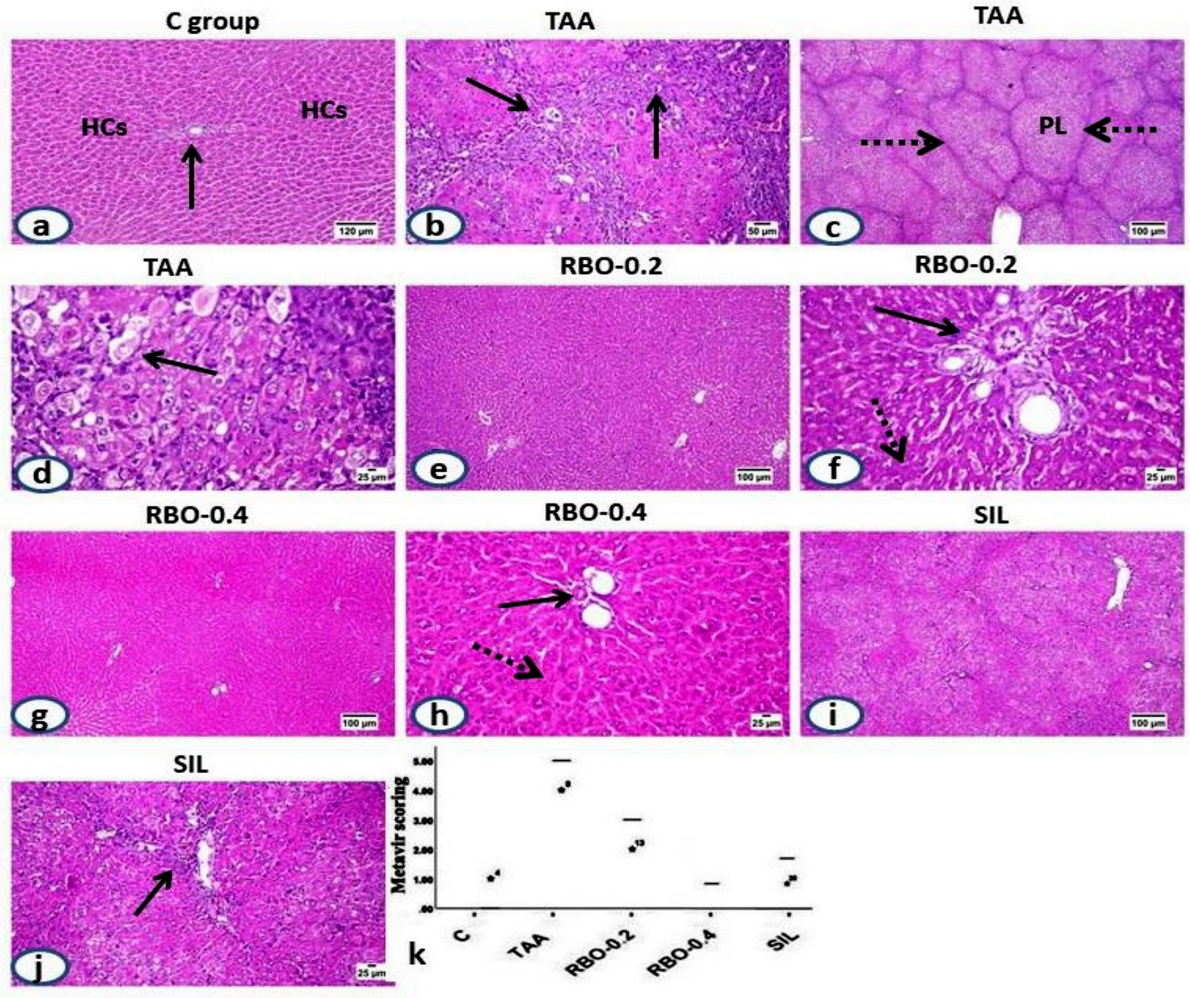
H&E stained liver sections; (a) Liver of control rat shows normal histological structure of central vein (arrow) and hepatic parenchymal cells (HCs). (b-d) liver of TAA-administrated rat showing; (b) thickening of the portal triad (arrow) with fibrous proliferation, inflammatory cells infiltration and proliferated bile duct epithelium, (c) extended fibrous septa (dotted arrow) with marked parenchymal pseudolobulation (PL), (d) vacuolar to ballooning degeneration of the hepatic cells (arrow) and marked apoptosis within pseudo-lobules. (f) (e–h) livers of RBO treated group showing marked retraction of fibrous proliferation without any evidence of pseudolobulation, only at low dose treatment (0.2 mL/rat), mild limited fibroplasia in the portal area (arrow) with scattered necrotic hepatocytes (dotted arrow) and scarce ones at the high dose (0.4 mL/rat) treatment. (I and j) Silymarin treated group showing moderate retraction of parenchymal fibrous with its limitation to the portal areas (arrow). (k) The scoring of the fibrosis extension in TAA and various treated groups using Metavir scoring scale. All data are presented as Mean± SEM, (n = 6). a *P*≤0.05 was assumed to denote statistical significance compared to the negative control, b *P*≤0.05 was assumed to denote statistical significance compared to TAA group. C: control; TAA: Thioacetamide; RBO: Rice bran oil.

The histological structure of portal regions, central veins, and hepatic parenchymal cells were normal in control rats’ livers **(Fig 5A)**, livers of rats given thioacetamide revealed significant fibroplasia, which began in the portal triads, accompanied by bile duct epithelial proliferation, vascular congestion, and mononuclear inflammatory cells infiltration **(Fig 5B)**, which showed peripheral extension toward the parenchyma as fibrous bands that resulted in marked parenchymal pseudo-lobulation **(Fig 5C)**. The hepatic cells within those pseudo-lobules showed vacuolar degeneration with eccentric nuclei, necrosis **(Fig 5D)**. Also, apoptosis along with inflammatory infiltrates was seen along the fibrous septa.

Regarding livers of various treated groups, variable degrees of retraction of fibrous proliferation were noticed, the best of which was detected in the RBO-treated group at the high dose (0.4 ml/rat). Livers of low dose RBO-treated (0.2 ml/rat) group **(Fig 5E and 5F)** revealed mild fibroplasia in the portal regions, along with bile duct epithelial proliferation and a few inflammatory cells infiltration. A moderate degree of hepatocellular degeneration, scattered necrosis, and apoptosis was seen. Livers of RBO-treated rats with a high dose (0.4 ml/rat) **(Fig 5G and 5H)** revealed scarce fibroplasia in the portal areas with minimal changes and good restoration of the hepatic cells.

Livers of Silymarin-treated rats **(Fig 5I and 5J)** exhibited moderate fibroplasia in the portal regions, with peripherally expanded incomplete septa, few inflammatory cells infiltration, and cholangiolar proliferation. A moderate degree of hepatocellular degenerative and necrotic alterations was noticed. The scoring of fibroplasia extension was evaluated using the Metavir scoring system (ranged from F0 = no fibrosis to F4 = cirrhosis) in all of the experimental groups **(Fig 5K)**.

**Fig 6:**
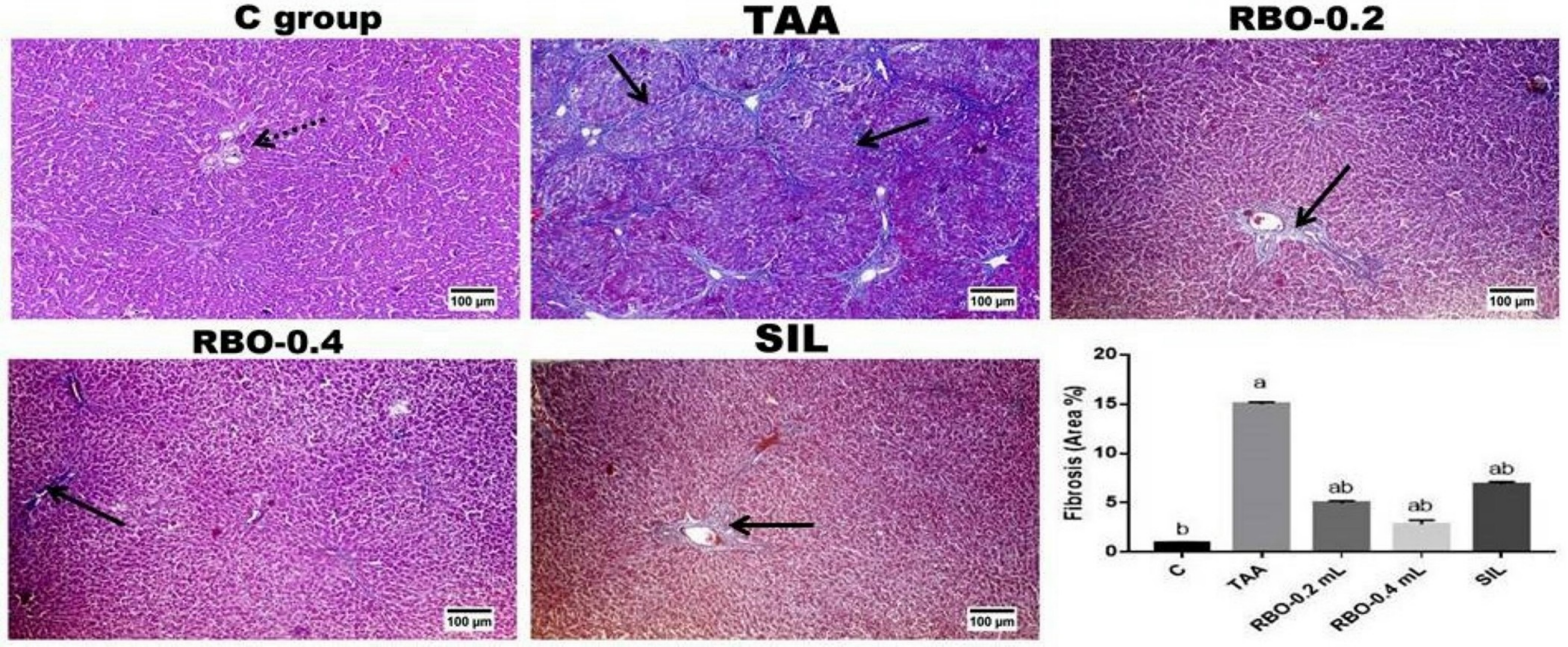
MTC stained liver sections. Control group showing normal limited amount of fibrous tissue in the portal areas (dotted arrow), the highest significant increase in MTC stained areas of fibrous tissue in the bridging fibrosis (arrow) in TAA group. While, RBO administration gave rise to a dose dependent significant decrease in MTC-stained areas with its limitation to portal triads (arrow) compared to TAA group and silymarin administrated group. The quantitative image analysis of the MTC stained areas presented as area percentage. All data are presented as Mean± SEM, (n = 6). ^a^ *P*≤0.05 was assumed to denote statistical significance compared to the negative control, ^b^ *P*≤0.05 was assumed to denote statistical significance compared to TAA group. C: control; TAA: Thioacetamide; RBO: Rice bran oil.

The Masson’s Trichrome stained liver sections of various groups’ revealed significant increased area percentage of fibroplasia in liver sections of thioacetamide group compared to the other groups. RBO administration at both low and high doses resulted in a dose-related significant decrease in fibrous tissue proliferation with its limitation to the portal areas compared to thioacetamide and silymarin administered groups. The latter group also revealed significant decrease in fibrous tissue proliferation compared to thioacetamide group **(Fig 6)**.

### Immunohistochemical investigation

**Fig 7:**
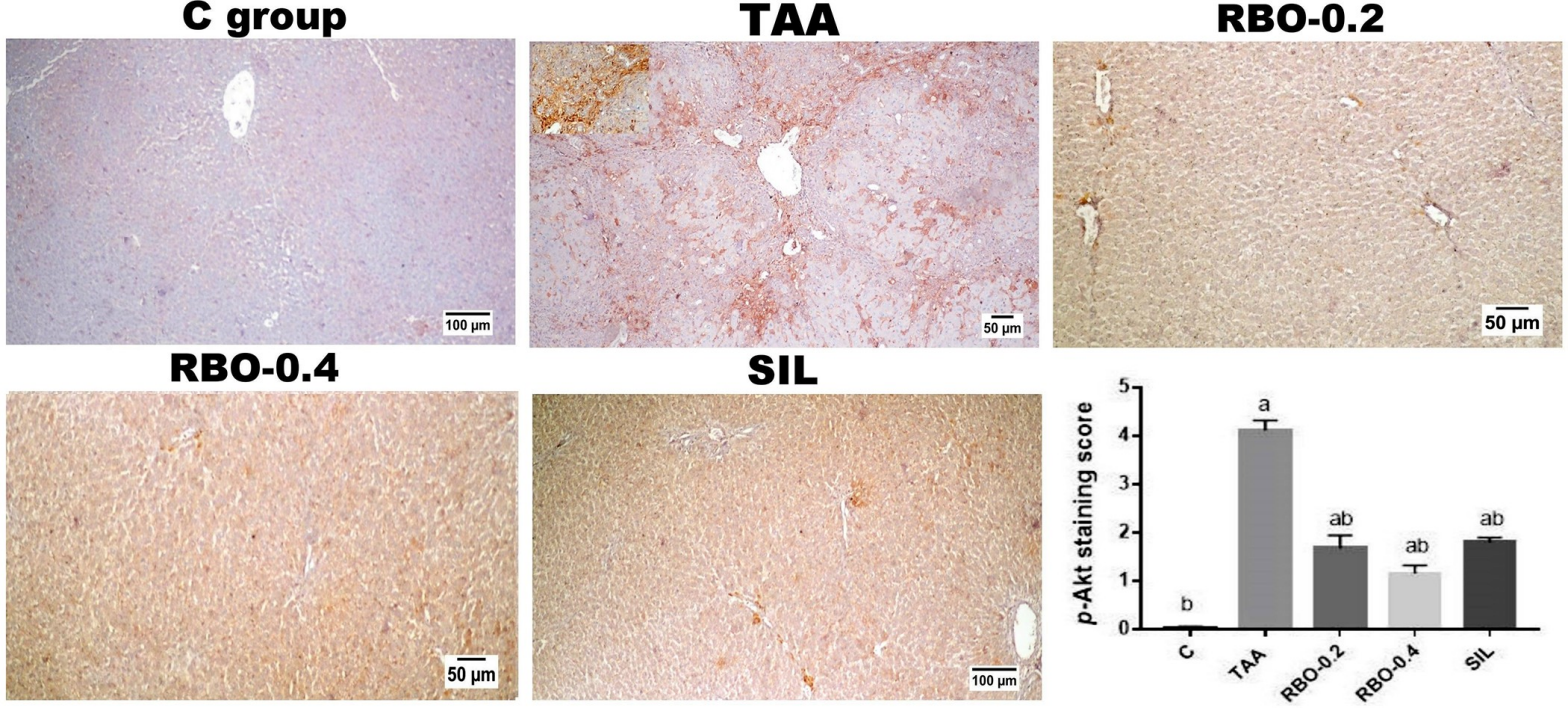
Immunohistochemical analysis of p-Akt expression in various experimental groups showing negative expression in control group, marked increased expression along the extended fibrous bands (upper insert) in TAA group, significant dose-related decreased expression following the decreased fibroplasia in RBO treated groups, and limited expression in SIL treated group. The quantitative image analysis of the area percent of the positive brown colour presented as intensity scores. All data are presented as Mean± SEM, (n = 6). ^a^ *P*≤0.05 was assumed to denote statistical significance compared to the negative control, ^b^ *P*≤0.05 was assumed to denote statistical significance compared to TAA group. C: control; TAA: Thioacetamide; RBO: Rice bran oil.

**Fig 8:**
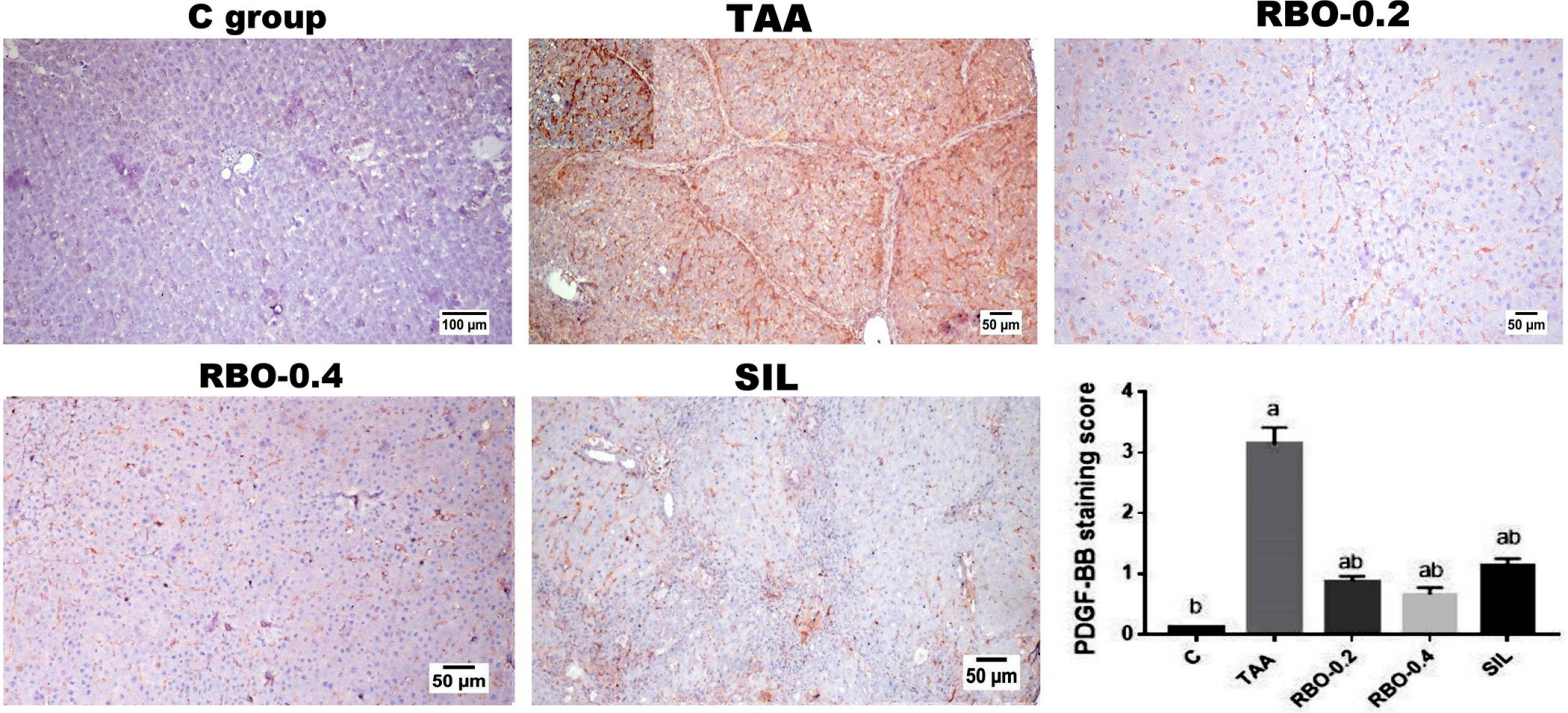
Immunohistochemical analysis of PDGF-BB expression in various experimental groups; showing negative expression in control group, significant increased along the fibrous septa and along the sinusoidal areas (upper insert) in TAA administrated group, significant dose related decreased expression in RBO which showing scattered expression in the sinusoidal areas, and mild expression along the incomplete septa as well as in sinusoidal areas in silymarin treated group. **(C)** Quantitative image analysis of the area percent of the positive brown colour presented as intensity scores. All data are presented as Mean± SEM, (n = 6). ^a^ *P*≤0.05 was assumed to denote statistical significance compared to the negative control, ^b^ *P*≤0.05 was assumed to denote statistical significance compared to TAA group. C: control; TAA: Thioacetamide; RBO: Rice bran oil.

As depicted in **(Figs 7 and 8)**, livers of the model fibrosis (TAA-induced) group showed marked expression of both p-Akt **(Fig 7)** and PDGF-BB **(Fig 8)** compared to the control group. While RBO-treated groups (0.2 and 0.4 mL/rat) two-week post-liver fibrosis induction showed marked dose-related deceased immuno-expression of p-Akt and PDGF-BB, particularly in the high dose treated group compared to the high dose TAA group and silymarin treated group. Silymarin treated group showed decreased expression of both markers. The quantitative analysis of the positive brown color of p-Akt and PDGF-BB, expressed as staining score, demonstrated positive (*p*≤0.05) overexpression in the TAA-treated group compared to the other treatment groups.

### Effects of RBO against TAA-induced NF- κβ and COX-2 activation

**Fig 9:**
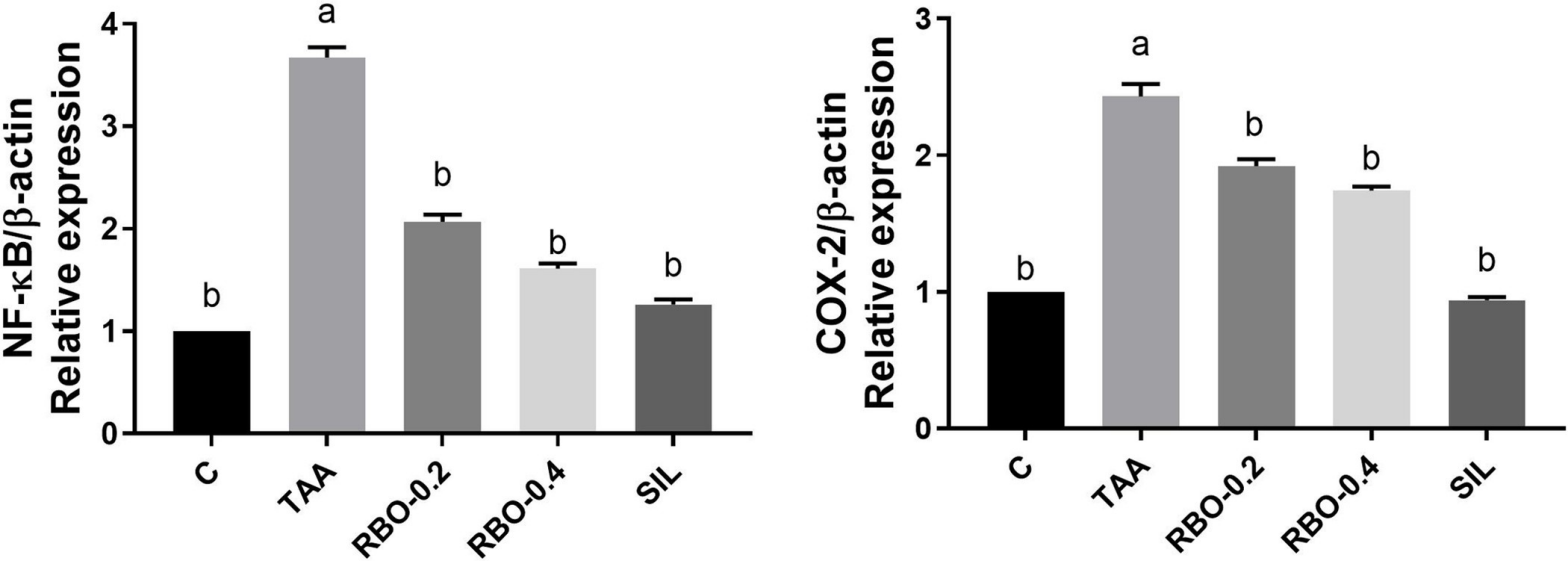
Effect of RBO on NF-κβ and COX-2 in TAA-induced liver fibrosis in rats. Control rats, treated with saline; TAA rats, treated with thioacetamide (100 mg/kg; three times per week for 2 weeks, *ip*); RBO rats, treated with TAA, RBO (0.2 and 0.4 mL/rat; daily for 2 weeks, *po*) and silymarin (100 mg/kg, *po*). All data are presented as Mean±SEM, (n = 6). ^a^ *P*≤0.05 was assumed to denote statistical significance compared to the negative control, ^b^ *P*≤0.05 was assumed to denote statistical significance compared to TAA group. TAA, Thioacetamide; RBO, Rice bran oil; NF- κβ, Nuclear factor kappa B; COX-2, Cyclooxygenase-2.

In this study, expression levels of NF-κβ and COX-2 inflammatory mediators in the liver were analyzed by qRT-PCR. The TAA-intoxicated group showed significant upsurges in the content of the mRNA of NF-κβ (3.67-fold) as compared to the negative control value (**Fig 9**). Treatment of TAA-intoxicated rats with silymarin, RBO (0.4 mL/rat), and (0.2 mL/rat) significantly downregulated hepatic NF- κβ expression (1.26, 1.61, and 2.07-fold) as compared to the TAA group. Similarly, TAA intoxication resulted in a substantial increase in hepatic COX-2 to 2.43-fold as compared to negative control values. Supplementation with silymarin, RBO (0.4 mL/rat), and (0.2 mL/rat) significantly restored COX-2 expression to 0.94, 1.74, and 1.92-fold **(Fig 9)**.

## Discussion

Thioacetamide (TAA) is an organosulfur compound that has been widely used to induce experimental liver injury and liver fibrosis. TAA gets metabolized into thioacetamide-S-oxide and acetamide immediately after administration to rats [2]. Thioacetamide-S-oxide, the metabolic intermediate of TAA, binds to certain macromolecules in the cell responsible for the change in cell permeability and interruption of calcium stores. It also inhibits mitochondrial activity eventually leading to cellular damage and hepatocyte necrosis [37, 38]. TAA is well-known hepatotoxin produces hardly reversible fibrosis in rodents similar to that of human, for that, it is an ideal model to test potential antifibrotic drugs [27]. Previous studies showed that TAA induced liver fibrosis through oxidative stress, evidenced by MDA elevation and suppression of GSH and SOD. TAA also upsurges the expression of α-SMA, TGF-β1, and PI3K/Akt pathway [39].

Rice bran oil (RBO) is rich in many bioactive phytochemicals such as γ-oryzanols, tocopherols, tocotrienols, carotenoids, phytosterols, squalene, policosanols, ferulic acid, and unsaturated fatty acids [13, 40]. Furthermore, it could exhibit hypocholesterolemic, hypolipidemic, anti-inflammatory, cytotoxic, and antioxidant effects. RBO contains nearly 38% oleic acid, 34% linoleic acid, and 18.6% palmitic acid [41].

Two weeks of TAA injection (100 mg/kg) three times weekly resulted in a significant increase in the activity of liver marker enzymes (ALT, AST) along with suppression in albumin and total proteins levels. Elevation of the activities of liver transaminases following TAA administration denotes liver cellular leakage and diminution in the structural and functional integrity of liver cells and is thus designated as an index of damage of the liver parenchyma cells [27, 42].

Our results implied that RBO administration significantly decreased serum transaminases, which is consistent with the previous findings of Rana et al. ‎[43], where RBO significantly decreased the activities of the enzymes of liver function test (AST, ALT, ALP) in N-nitrosodiethylamine-induced oxidative stress in rats.

Serum levels of albumin and total proteins reflect the functional status of hepatocytes [44]. TAA poisoning causes ubiquitin-associated protein degradation, which might be one of the main reasons for a drop in total protein levels in the blood. TAA treatment resulted in substantial reductions in serum total protein, albumin levels, and the A/G ratio in the current investigation. Hypoproteinemia might also be caused by an inflammatory response or a disruption in protein production in the fibrotic liver. Furthermore, free radicals and toxic metabolites generated by TAA are responsible for cellular death and the liver’s incapability to execute its metabolic and excretory functions [1].

Compared to the positive control group, the RBO adjusted A/G ratio and considerably boosted both the total protein and the albumin levels. These results are consistent with those obtained by Abd Allah et al. [45], who cited that RBO normalized serum albumin, globulins, total proteins, and A/G ratio, owing to its hepatoprotective activity.

TAA-induced liver fibrosis is exacerbated by oxidative damage, which has been identified as a key molecular cause of TAA-induced hepatotoxicity. Oxidative agents and lipid peroxidation products aid the production of profibrogenic growth factors, cytokines, and prostaglandins. Additionally, reactive oxygen species (ROS), which include the epithelium, activated inflammatory cells, and/or microvascular endothelium, contribute to liver injury [46]. GSH is a non-enzymatic antioxidant found in hepatocytes that shield the membrane protein thiols of liver cells from the ROS damaging effects such as hydrogen peroxide and superoxide radicals. When GSH is reduced due to oxidation, it is transformed to glutathione disulfide, which causes lipid peroxidation [44].

In this work, rats treated with TAA had substantially lower hepatic GSH content, restored by RBO or silymarin treatment. Furthermore, as compared to the negative control group, TAA injection enhanced liver MDA content. When compared to the TAA-treated group, MDA content was substantially lower in the RBO or silymarin-treated groups. These findings are consistent with Al-Okbi et al. [13]; they claimed that RBO might successfully protect against liver inflammation and oxidation caused by a high-fat diet, as seen by MDA and (Tumor necrosis factor-α) TNF-α reductions.

In the current study, hepatic TNF-α, IL-1β, NF-κβ, Collagen I, CTGF, and Hydroxyproline levels in the TAA group were significantly elevated, compared to the negative group.

Tumor necrosis factor-α is a multifunctional cytokine contributing to chronic progress of liver inflammation that accounts for liver fibrosis. TNF-α causes the activation of local HSCs into fibrogenic myofibroblasts during the inflammatory phase [1]. Moreover, IL-1β, a member of the interleukin-1 superfamily generated by the liver, leads to the activation of resident immune cells and the migration of other leukocytes to the injured liver, resulting in chronic inflammation, along with pro-inflammatory IL-6 and TNF-α.

Compared to the positive control group, treatment of RBO at both dosage levels (0.2 and 0.4 mL/rat) for two weeks after development of liver fibrosis made a considerable reduction in liver content of TNF-α, IL-1β, and NF-κβ. Furthermore, tocotrienol, a component of RBO, has been shown to block the inflammatory transcription factor NF-κβ, which is connected to inhibiting chronic inflammation, pro-fibrotic cytokines, apoptosis, and oxygen-free radical-induced damage [47].

The pro-inflammatory cytokine regulator, NF-κβ, is suggested to be a master contributor to liver inflammation and fibrogenesis processes through regulation of pro-inflammatory cytokines production and HSCs activation [48]. In normal conditions, NF-κβ is sequestered in the cytoplasm by the inhibitory protein I- κβ, which inhibits NF-κβ from nuclear translocation. Upon liver injury, I-κB undergoes phosphorylation and degradation; thus, NF-κβ is activated and translocated into the nucleus, where it induces the transcription of hundreds of target genes involved in the regulation of inflammatory and immune responses [49].

NF-κβ pathway activation has been reported to be associated with enhanced liver fibrogenesis through the stimulation of pro-inflammatory mediators such as TNFα, IL-6, iNOS, COX-2, PGE2, and MMP-9 [50]. Our findings are in agreement with previously reported studies that TAA activated NF-κβ. However, RBO treatment significantly modulated TAA-induced NF-κβ upregulation.

Interestingly, the phytoconstituent γ-oryzanol from RBO, repressed NF-κβ initiation and downregulated the inflammatory responses of the macrophage cell line [13, 51].

COX-2 is one of the NF-κβ downstream inflammatory targets induced by various stimuli, including oxidative stress, inflammation, and carcinogens [52]. Previous studies indicated that COX2 activation with the subsequent PGs release favors the development of the necroinflammatory condition, HSCs proliferation, and angiogenesis that could contribute to hepatic fibrosis and carcinogenesis [53]. Thereby, the mechanism of some antifibrotic and antiproliferative agents such as celecoxib is mediated via inhibiting COX-2 in HSCs with a consequent reduction in PGs [53]. Our results showed that RBO treatment significantly decreased mRNA expression of COX-2 and TGF-β1, as well as reduced hepatic collagen deposition compared to TAA-treated rats. These findings suggest RBO as an antifibrotic agent of hepatic fibrosis.

Abnormal synthesis and accumulation of type I collagen in the extracellular matrix is considered the end product of fibrosis, produced by activated stellate or Ito cells in the damaged liver. Furthermore, hydroxyproline, a characteristic amino acid present in collagen, is the major component of the collagen triple helix. It reflects the total collagen content in the liver tissue. Hydroxyproline is used as a marker to determine the degree of fibrosis and assess the efficacy of new antifibrotic drugs [54].

Liver hydroxyproline content was markedly amplified in the TAA intoxicated group compared to the negative control group (*p*≤0.05). Similarly, collagen I was significantly upregulated in the livers of rats in the TAA administered group compared with the negative control group (*p*≤0.05).

RBO revealed a significant reduction of liver collagen I as compared to the fibrotic group.

Supporting our findings, Phetpornpaisan et al. ‎[55] mentioned that the ethanolic extract of rice bran is rich in bio-active elements including caffeic acid, cyanidin-3-glucoside, ferulic acid, and p-coumaric acid, according to their study. Furthermore, rice bran extract demonstrated antioxidation, MMP-2 and MMP-9 inhibition, wound healing benefits, as well as the immunomodulatory effect.

Additionally, HSCs and hepatocytes produce “connective tissue growth factor (CTGF, also known as CCN2)”, which is highly expressed during liver fibrosis. CTGF, a robust fibrotic activity, is a key regulator of the TGF-β1 activator, increasing the cytokine’s fibrogenic effects, especially in the hepatic cells and other places. Moreover, hepatocytes synthesize CTGF in the damaged liver; and TGF‐β1 stimulated hepatocytes to appear to be the principal cellular source of CTGF in the liver [19]. Moreover, a positive correlation between gene expression of TGF-β1, CTGF, and α-SMA supports their involvement in the fibrogenic mechanism. TGF-β1 is the most potent inducer of CCN2; additionally, CCN2 is known to act downstream of the TGF-β1 signaling pathway. The promoter activity CCN2 is upregulated not only by TGF-β1 but also by PDGF, ethanol, and acetaldehyde [56].

Rats treated with RBO after induction of liver fibrosis showed a significant reduction of liver content of hydroxyproline, collagen I, and CTGF compared to the positive control group. RBO is rich in antioxidant phytoconstituents such as tocotrienol, tocopherols, and many others that could protect against hepatic injury. Balah et al. ‎[57] reported that vitamin E inhibited cyclosporin A (CsA)-induced TGF-β1/Smad signaling pathway and subsequent suppression of CTGF and TIMP-1 expression in rat liver.

In the fibrotic liver, TGF-β1 is secreted by both autocrine and paracrine cells. TGF-1 stimulates collagen, α-SMA, and other ECM proteins transcription and release [58]. Confirming this, TGF-β1 and α-SMA expression were substantially increased in TAA-intoxicated rats.

The cytoplasmic protein tyrosine kinase FAK is a non-receptor cytoplasmic protein tyrosine kinase. When cells connect to ECM proteins through integrin binding, FAK is activated. Integrins are the principal adhesion receptors that transfer signals between ECM pathways and cytoplasmic domains across the cell plasma membrane. FAK is also activated in response to TGF-β1 activation and also other cytokines and growth factors [9, 59]. Consistent with the current study’s findings, TGF-β1 activates HSCs to produce fibronectin, α-SMA, and CTGF, markers of HSC activation. Therefore, FAK activation in fibrotic liver tissue is associated with increased α-SMA and collagen production [59]. Similarly, active FAK increases invasion and myofibroblast development and resistance to apoptosis in chronic liver disorders, suggesting its contribution to liver fibrosis [9].

## Conclusion

Throughout the four-week of the current investigation, RBO displayed beneficial protective therapy against TAA-induced liver fibrosis. RBO amended oxidation-induced damage via dropping down of lipid peroxidation and elevating GSH. Furthermore, RBO substantially curtailed inflammation by regulation of TNF-α, IL-6, NF-κβ, COX2, pAKT, PDGF which contributes to the downregulation of the TGF-β1 pathway. Most significantly, RBO inhibited the TGF-β1/FAK signaling pathway, which reduced HSC growth and division. Finally, RBO inhibited ECM deposition and fibrosis progression by suppressing fibrogenic factors expression as TGF-β1, α-SMA, collagen I, CTGF, and PDGF. More research is warranted to understand the connection between RBO and the TGF-β1/FAK pathway in the prevention of liver fibrosis, as well as additional pathways that mediate RBO’s antifibrotic activity.

## Supporting information

S1 File (DOCX)

S2 File (DOCX)

S1 Graphical abstract (PNG)
