# Supplementary material for: The involvement of TGF-β1 /FAK/α-SMA pathway in the antifibrotic impact of rice bran oil on thioacetamide-induced liver fibrosis in rats
Source: PLoS One. 2021 Dec 29;16(12):e0260130. doi: 10.1371/journal.pone.0260130 (PMC8716044; doi:10.1371/journal.pone.0260130)
Supplement: S1 File — (DOCX) [file pone.0260130.s001.docx]

**RBO supplementary file**

**(Data)**

| **Gp.** | **Serum** | | | | |
| --- | --- | --- | --- | --- | --- |
|  | **ALT**  **(U/L)** | **AST (U/L)** | **Total protein (g/dL)** | **Albumin (g/dL)** | **A/G ratio** |
| **Negative control** | 25.95 | 40.36 | 6.57 | 3.32 | 1.02 |
|  | 20.01 | 38.91 | 6.87 | 3.16 | 0.85 |
|  | 29.72 | 44.14 | 7.72 | 4.88 | 1.72 |
|  | 25.36 | 40.83 | 6.62 | 3.46 | 1.10 |
|  | 21.75 | 36.84 | 6.83 | 3.46 | 1.03 |
|  | 23.64 | 36.00 | 6.00 | 3.10 | 1.07 |
| **Positive control (TAA)** | 27.50 | 57.69 | 4.92 | 2.11 | 0.75 |
|  | 28.58 | 45.04 | 4.54 | 2.09 | 0.85 |
|  | 34.38 | 43.44 | 3.91 | 1.40 | 0.56 |
|  | 30.20 | 44.73 | 4.78 | 2.05 | 0.75 |
|  | 29.71 | 48.08 | 4.03 | 1.60 | 0.65 |
|  | 28.96 | 43.79 | 4.96 | 1.20 | 0.32 |
| **RBO (0.2 mL/rat)** | 18.18 | 39.23 | 4.56 | 2.91 | 1.77 |
|  | 18.75 | 35.61 | 5.90 | 2.45 | 0.71 |
|  | 17.30 | 38.38 | 6.16 | 3.75 | 1.55 |
|  | 22.22 | 42.68 | 6.94 | 3.53 | 1.04 |
|  | 15.64 | 39.73 | 5.85 | 2.72 | 0.87 |
|  | 14.00 | 35.00 | 5.00 | 2.96 | 1.45 |
| **RBO (0.4 mL/rat)** | 20.09 | 37.77 | 5.75 | 3.30 | 1.35 |
|  | 17.59 | 35.29 | 4.69 | 2.33 | 0.99 |
|  | 16.91 | 36.94 | 6.07 | 3.43 | 1.30 |
|  | 12.10 | 37.85 | 6.74 | 3.84 | 1.32 |
|  | 12.58 | 38.19 | 6.63 | 3.68 | 1.25 |
|  | 15.00 | 35.30 | 6.00 | 2.86 | 0.91 |
| **Silymarin (100 mg/kg)** | 31.53 | 49.13 | 5.57 | 3.13 | 1.28 |
|  | 29.18 | 41.63 | 4.97 | 2.51 | 1.03 |
|  | 27.65 | 44.71 | 5.51 | 2.93 | 1.13 |
|  | 26.17 | 45.75 | 4.93 | 2.56 | 1.08 |
|  | 21.86 | 42.37 | 5.36 | 2.74 | 1.04 |
|  | 28.00 | 40.18 | 4.34 | 2.75 | 1.72 |

| **Gp.** | **Liver homogenate** | |
| --- | --- | --- |
|  | **GSH (μMol/g tissue)** | **MDA (nMol/g tissue)** |
| **Negative control** | 20.46 | 52.00 |
|  | 24.94 | 62.35 |
|  | 21.74 | 54.35 |
|  | 20.02 | 53.00 |
|  | 23.07 | 57.68 |
|  | 23.08 | 57.70 |
| **Positive control (TAA)** | 11.91 | 119.14 |
|  | 10.91 | 111.00 |
|  | 12.74 | 127.44 |
|  | 13.87 | 138.73 |
|  | 12.13 | 121.30 |
|  | 11.00 | 121.00 |
| **RBO (0.2 mL/rat)** | 16.74 | 41.86 |
|  | 15.13 | 39.00 |
|  | 15.65 | 39.13 |
|  | 17.36 | 43.41 |
|  | 17.63 | 44.08 |
|  | 14.90 | 39.00 |
| **RBO (0.4 mL/rat)** | 19.69 | 49.22 |
|  | 18.54 | 46.34 |
|  | 18.60 | 57.00 |
|  | 18.26 | 45.64 |
|  | 18.60 | 48.00 |
|  | 21.33 | 53.32 |
| **Silymarin (100 mg/kg)** | 16.06 | 41.00 |
|  | 23.15 | 57.88 |
|  | 23.43 | 58.57 |
|  | 24.63 | 63.00 |
|  | 20.02 | 50.05 |
|  | 17.51 | 45.00 |

| **Gp.** | **Liver Homogenate** | | | | | | | | |
| --- | --- | --- | --- | --- | --- | --- | --- | --- | --- |
|  | **TNF-α (ng/L)** | **IL-1β (pg/mL)** | **NF-κβ (pg/mL)** | **TGF-β (pg/mL)** | **Collagen I (ng/mL)** | **CTGF (pg/mL)** | **α-SMA (ng/mL)** | **Hydroxyproline (ng/mL)** | **FAK (pg/mL)** |
| **Negative control** | 232.03 | 246.43 | 3651.67 | 4572.70 | 28.23 | 1345.92 | 449.24 | 2958.59 | 10684.25 |
|  | 316.99 | 304.21 | 4469.10 | 5398.89 | 39.71 | 1583.34 | 434.21 | 2601.86 | 10998.18 |
|  | 250.19 | 216.32 | 2925.66 | 5949.69 | 32.63 | 1395.90 | 577.56 | 3329.86 | 10718.76 |
|  | 232.93 | 331.87 | 4117.02 | 3971.83 | 31.35 | 1970.71 | 577.80 | 2799.22 | 10690.95 |
|  | 305.49 | 251.75 | 2871.16 | 3471.11 | 28.00 | 1814.51 | 491.90 | 3499.30 | 9051.24 |
|  | 308.12 | 265.85 | 3721.24 | 5098.46 | 35.20 | 1333.43 | 406.47 | 3401.20 | 10273.75 |
| **Positive control**  **(TAA)** | 801.88 | 790.54 | 23651.44 | 6800.91 | 61.32 | 2033.19 | 760.78 | 5031.10 | 14587.68 |
|  | 989.06 | 649.81 | 33894.93 | 6775.88 | 65.66 | 2126.90 | 852.85 | 4680.97 | 14776.96 |
|  | 785.77 | 918.75 | 28833.39 | 6725.81 | 69.90 | 2176.89 | 737.00 | 5822.50 | 13978.36 |
|  | 696.62 | 666.80 | 22920.44 | 8033.46 | 48.04 | 1770.78 | 753.78 | 4821.52 | 16368.74 |
|  | 670.73 | 857.07 | 24580.45 | 9655.03 | 61.22 | 2126.90 | 871.62 | 4248.44 | 15635.32 |
|  | 961.20 | 737.19 | 21459.15 | 10856.76 | 83.41 | 1995.70 | 892.71 | 4342.08 | 14786.51 |
| **Rice bran oil low**  **(0.2 mL/rat)** | 437.78 | 249.84 | 4832.32 | 4497.59 | 39.19 | 1908.23 | 709.50 | 2549.51 | 12686.81 |
|  | 395.96 | 243.23 | 4141.81 | 6800.91 | 46.17 | 1508.37 | 545.86 | 4382.21 | 11942.55 |
|  | 265.22 | 385.26 | 4797.54 | 4898.17 | 43.09 | 1589.59 | 573.60 | 4198.23 | 14155.79 |
|  | 441.56 | 345.94 | 4893.33 | 6827.40 | 35.40 | 1645.82 | 607.28 | 3907.23 | 13460.45 |
|  | 519.21 | 271.19 | 7058.51 | 5482.67 | 34.24 | 1745.78 | 530.36 | 3675.35 | 12598.24 |
|  | 476.07 | 315.86 | 3493.04 | 6905.39 | 32.47 | 1602.08 | 732.11 | 3531.50 | 6766.69 |
| **Rice bran oil high**  **(0.4 mL/rat)** | 456.35 | 281.88 | 5284.52 | 4372.41 | 37.78 | 1683.31 | 492.36 | 3637.36 | 11142.67 |
|  | 269.66 | 331.38 | 3788.80 | 4176.46 | 38.13 | 1364.66 | 786.65 | 3320.94 | 14739.87 |
|  | 398.17 | 271.68 | 4936.67 | 5824.51 | 23.99 | 1702.05 | 488.40 | 4037.70 | 10216.55 |
|  | 351.50 | 264.40 | 3354.08 | 5849.54 | 46.59 | 1439.64 | 654.83 | 2834.89 | 9718.48 |
|  | 354.13 | 320.71 | 4076.17 | 4409.00 | 37.49 | 1577.09 | 601.45 | 2469.25 | 10790.37 |
|  | 406.14 | 284.31 | 4232.43 | 4802.36 | 34.40 | 1620.83 | 635.14 | 2527.22 | 10620.92 |
| **Silymarin**  **(100 mg/kg)** | 392.18 | 451.77 | 4208.17 | 5126.38 | 35.69 | 1876.99 | 717.49 | 3178.25 | 10465.88 |
|  | 328.49 | 386.73 | 3755.62 | 6446.08 | 33.14 | 1670.81 | 641.08 | 4465.77 | 11958.53 |
|  | 385.28 | 339.15 | 4700.21 | 4871.69 | 43.21 | 1639.57 | 736.30 | 3419.04 | 10757.13 |
|  | 325.79 | 492.05 | 4214.77 | 4873.13 | 29.03 | 1695.80 | 698.54 | 3777.91 | 13506.81 |
|  | 321.43 | 255.18 | 5286.12 | 5496.58 | 44.86 | 1539.60 | 551.80 | 3450.25 | 11902.90 |
|  | 262.35 | 325.57 | 4055.12 | 5295.86 | 30.49 | 1608.33 | 537.93 | 3905.08 | 12746.58 |
