# Supplementary material for: The involvement of TGF-β1 /FAK/α-SMA pathway in the antifibrotic impact of rice bran oil on thioacetamide-induced liver fibrosis in rats
Source: PLoS One. 2021 Dec 29;16(12):e0260130. doi: 10.1371/journal.pone.0260130 (PMC8716044; doi:10.1371/journal.pone.0260130)
Supplement: S2 File — (DOCX) [file pone.0260130.s002.docx]

| **The ARRIVE guidelines 2.0** | |
| --- | --- |
| **a.** After one-week of acclimatization, rats were divided into five groups, each with six animals, according to the following scheme: Group 1: the negative control group; rats were injected intraperitoneally (ip) with saline three times per week for two successive weeks, Group 2: the positive thioacetamide control (TAA) group; rats were ip injected with TAA (100 mg/kg) three times per week for two successive weeks to provoke liver fibrosis according to Wallace et al. (2015) with some modification based on our preliminary studies. Group 3 and 4: treatment groups; rats received orally rice bran oil (RBO; 0.2 and 0.4 mL/kg, orally) daily for 2 weeks, after three times per week of TAA injection for two successive weeks. Group 5: reference group; rats received silymarin (100 mg/kg, orally), daily for 2 weeks after 2 weeks of TAA injection three times per week.  **b.** Thirty adult male Wistar rats, five months of age, weighing 150-200 g, were procured from the “Animal House Colony at the National Research Centre (NRC, Egypt)”. Rats were kept in plastic cages at a room temperature of 25 °C, with a 12 h light and 12 h dark cycle. | **1-Study design** |
| 1. A total of thirty adult rats were divided into five groups, each with six animals were used in the experiment. 2. According to a previous pilot study and previous researches performed within our lab , the sample sized was determined. | **2- Sample size** |
| 1. **Inclusion criteria**  - Adult male male Wistar rats 150-200 g - Weight - Five months of age - No comorbid diseases  1. **b. Exclusion criteria**  - comorbid diseases - aged rats - Female rats | **3- Inclusion and exclusion criteria** |
| Experimental animals were randomly allocated into different study groups and coded using a ‎serial numbering system. | **4- Randomisation** |
| Animal samples were treated blindly during the investigational process.‎ | **5- Blinding** |
| **GC-MS chromatogram analysis**  Analysis of rice bran chemical constituents  **Determination of liver function**  Serum activities of aspartate aminotransferase (AST) and alanine aminotransferase (ALT).  **Determination of oxidative stress biomarkers**  Liver contents of reduced glutathione (GSH), Malondialdehyde (MDA).  **Determination of pro-fibrotic and inflammatory markers**  Liver contents of tumor necrosis factor-alpha (TNF-α) , nuclear factor-kappa B (NF-κB), interleukin 1 beta (IL-1β), transforming growth factor β1 (TGF-**β1),** alpha-smooth muscle actin (α-SMA), collagen I, hydroxyproline, connective tissue growth factor (CTGF) and focal adhesion kinase (FAK)  **Histopathological examination**  Light microscope (Olympus, Germany) was used to examine the obtained slides stained by H&E. Fibrosis was assessed using the Metavir grading method (ranged from F0=no fibrosis to F4=cirrhosis)  **Immunohistochemical studies**  PDGF-BB and p-AKT liver content were detected using "avidin-biotin-peroxidase. Quantification of the positive brown area of each marker’s expression was implemented as an optical density in 7 high-power microscopic fields using image analysis software (Image J, 1.46a, NIH, USA).  **Comparative RT-qPCR**  Quantification of the target genes (NF-κB, COX-2, and β-actin) expression in liver tissues. | **6-Outcome measures** |
| **a.** The values are expressed as mean ± standard error of six observations in each group. All groups were subjected to one-way analysis of variance (ANOVA), which was followed by Tukey's multiple comparisons test to determine the intergroup variability by using Graphpad Prism® software, version 8 (Inc., San Diego, USA).  **b.** When the difference was *p*≤0.05, it was judged significant. | **7- Statistical methods** |
| Healthy adult male Wistar rats, of five months of age, weighing 150-200 g, were obtained from the “Animal House Colony at the National Research Centre (NRC, Egypt)”. | **8- Experimental animals** |
| **Experimental design**  After a one-week of acclimatization, rats were divided into five groups, each with six animals, according to the following scheme: Group 1: the negative control group; rats were injected intraperitoneally (*ip*) with saline three times per week for two successive weeks, Group 2: the positive control (TAA) group; rats were *ip* injected with TAA (100 mg/kg) three times per week for two successive weeks to provoke liver fibrosis according to Wallace et al. with some modification based on our preliminary studies. Group 3 and 4: treatment groups; rats received orally RBO (0.2 and 0.4 mL/kg, orally) daily for 2 weeks after 2 weeks of TAA injection. Group 5: reference group; rats received silymarin (100 mg/kg, orally), daily for 2 weeks after 2 weeks of TAA injection.  **Preparation of blood and tissue samples**  At the end of the experiment, blood samples were collected from the retro-orbital venous plexus of each rat under mild ketamine anesthesia. Serum samples were extracted from the blood samples and stored at -20 °C for subsequent biochemical analysis. Rats were euthanized by cervical dislocation directly after blood sample, and livers were quickly removed, cleaned in ice-cold saline, blotted dry, and weighed. The left lobe of each rat's liver was dissected out and put in 10% buffered neutral formalin for histopathological and immunohistochemical examinations, while another weighted portion was preserved frozen at -80 °C for subsequent molecular and biochemical studies.  To create a 20 percent w/v homogenate, the later weighed component of each hepatic tissue was homogenized with ice-cooled saline using a homogenizer “Medical equipment, MPW-120, Poland”. To eliminate cell debris, the homogenate was centrifuged at 4000 rpm for 5 minutes at 4 °C in a cooling centrifuge “Laborzentrifugen, 2k15, Sigma, Germany”. Aliquots were then stored at -80 °C for biological examination. | **9- Experimental procedures** |
| **GC-MS chromatogram of RBO analysis**  GC/MS analysis of rice bran oil consists of 10 compounds. The total peak areas of the detected compounds are 100%.  The major compounds are Cyclooctane, butyl­ (13.17%), 9­Tricosene, (Z)­ (CAS) (18.98%), 1­Octadecanol (CAS) (15.43%) and 1­Heptadecene (CAS) (9.30%) for which represented (56.88%), of the total peak areas. The identification was accomplished using computer search user-generated reference libraries, incorporating mass spectra. Peaks were examined by single-ion chromatographic reconstruction to confirm their homogeneity. In some cases, when identical spectra have not been found, only the structural type of the corresponding component was proposed on the basis of its mass spectral fragmentation. Reference compounds were co-chromatographed, when possible, to confirm GC retention times.  **Liver transaminases**  Intraperitoneal injection of TAA (100 mg/kg) three times weekly for two weeks markedly (p≤0.05) increased liver transaminases (ALT; 29.9±0.98 U/L and AST; 47.1±2.22 U/L) in serum, showing a percent of elevation (22.5% and 19.24%, respectively) as compared to the negative control group (ALT; 24.4±1.4 U/L and AST; 39.5±1.21 U/L). Groups treated with RBO (0.2 and 0.4 ml/rat) for two weeks after inducing liver fibrosis showed a substantial (P≤0.05) reduction of liver enzymes (ALT; 17.7±1.15 and 15.7±1.26 U/L and AST; 38.4±1.167 and 36.9±0.53 U/L) showing the percent of reduction of 40.8% and 47.5% for ALT, 18.47% and 21.65 for AST, respectively. Notably, RBO improves liver enzymes level in a dose corresponding manner. This effect was better than that of the reference drug, silymarin, which showed no significant change (p≤0.05) (ALT; 27.4±1.33 U/L and AST; 44.0±1.33 U/L) as compared to the positive control group.  **Serum levels of total protein, albumin, and A/G ratio**  Intraperitoneal injection of TAA (100 mg/kg) three times weekly for two weeks noticeably (p≤0.05) decreased in serum total protein (4.5±0.19 g/dL), albumin concentration (1.7±0.16 g/dL) showing a percent of reduction (33.82% and 52.78%) respectively as compared to the negative control group (6.8±0.23, 3.6±0.27 g/dL), as well as a reduction of the A/G ratio (0.65±0.08) as compared to the negative control group (1.13±0.12). Rat groups treated with RBO at both tested dose levels (0.2 and 0.4 mL/rat) for two weeks post-induction of the liver, revealed a substantial increase in serum total protein level (1.23±0.23 g/dL and 1.19±0.08, respectively), serum albumin (3.1±0.2 g/dl and 3.2±0.23 g/dL, respectively) as compared to the positive control group. As well, groups treated with RBO (0.2 and 0.4 mL/rat) and silymarin (100 mg/dL) showed normal A/G ratio (1.23±0.17, 1.19±0.08, and 1.21±0.11, respectively), as compared to the negative control group.  **Effect on oxidative stress indicators in liver tissue**  TAA administration resulted in substantial (p≤0.05) diminution of hepatic GSH content by 45.49% but was rescued by RBO or silymarin administration. GSH was significantly increased in rat group-administered RBO (0.4 mL/rat) by 58.67%, compared to the TAA-treated group. Data revealed an upsurge in MDA level in the control positive group as compared to the negative control group by 119.04%. However, MDA levels were considerably lower in the RBO-treated groups (0.4 mL/rat) by 59.46%, compared to the TAA-treated group.  **Proinflammatory cytokines (TNF-α and IL-1β) and proinflammatory cytokine regulator (NF-****κB)**  Intraperitoneal injection of TAA (100 mg/kg) thrice weekly for 2 weeks noticeably (p≤0.05) increased TNF-α, IL-1β and NF-κβ (817.5±54.00 ng/mL, 770.0±43.32 pg/mL and 25890.0±1896.66 pg/mL, respectively) in hepatic tissue homogenate as compared to negative control group (TNF-α; 274.3±16.35 ng/mL, IL-1β; 269.4±17.11 pg/mL and NF-κβ; 3626.0±259.61 pg/mL), showing a percent of elevation (198.0%, 185.8% and 85.99%), respectively. Groups treated with RPO at both tested levels (0.2 & 0.4 ml/rat) for two weeks post-induction of liver fibrosis significantly (P≤0.05) decreased TNF-α (422.6±35.71 and 372.7±25.93 ng/L), with percent of reduction (48.31% & 54.41%) respectively, IL-1β (301.9±23.22 and 292.4±11.12 pg/ml) respectively and showing a percent of reduction (60.79% and 62.02%) and NF-κB (4869.4±490.68 and 4278.8±293.41 pg/mL, respectively) and showing a percent of reduction (81.2% and 83.47%, respectively) as compared to control positive group. Groups treated with RBO at both tested levels (0.2 and 0.4 mL/rat) showed no significant change from the group treated with silymarin in all inflammatory parameters (TNF-α; 335.9±19.48 ng/mL, IL-1β; 375.1±35.51 pg/mL and NF-κβ; 4370.0±221.75 pg/mL) that showed the percent of reduction (TNF-α; 58.92%, IL-1β; 51.28% and NF-κβ; 83.12%).  **Liver fibrosis biomarkers (Hydroxyproline, Collagen I, and CTGF)**  TAA injection (100 mg/kg, ip) thrice weekly for 2 weeks noticeably (p≤0.05) increased hydroxyproline, collagen I and CTGF (4824.4±232.66 ng/mL, 64.9±4.76 ng/mL and 2038.4±60.09 pg/mL, respectively) in tissue homogenate, as compared to negative control group (3098.3±148.50 ng/mL, 32.5±1.82 ng/mL and 1574.0±109.08 pg/mL, respectively), showing a percent of elevation (35.78%, 49.92% and 22.78%, respectively). Groups treated with RBO (0.2-0.4 mL/rat) for two weeks post-induction of liver fibrosis showed a significant (p≤0.05) reduction of liver hydroxyproline (3707.3±265.13 and 3137.9±258.49 ng/mL), collagen I (38.4±2.19 and 36.4±2.99 ng/mL) and CTGF (1666.6±57.80 and 1564.6±55.34 pg/mL) as compared to control positive group and showing % of reduction in hydroxyproline (23.15% and 34.96%), collagen I (40.83% and 43.91%) and CTGF (18.24% and 23.24%). Groups treated with RBO (0.4 mL/rat) showed no considerable change with the group treated with silymarin hydroxyproline, collagen I and CTGF (3699.4±186.91 ng/mL, 36.1±2.69 ng/mL and 1671.9±46.63 pg/mL, respectively) with % of reduction of hydroxyproline, collagen I and CTGF (23.31%, 44.38% and 44.38%, respectively).  **TGF-β1 /FAK/****α-SMA pathway**  TAA injection (100 mg/kg, ip) thrice weekly for 2 weeks noticeably (p≤0.05) increased TGF-β1, FAK and α-SMA (4743.8±376.56 pg/mL, 15022.3±345.60 pg/mL and 811.5±27.91 ng/mL, respectively) in tissue homogenate, as compared to negative control group (4743.8±376.56 pg/mL, 10402.9±286.35 pg/mL and 489.5±30.07 ng/mL, respectively), showing a percent of elevation (41.73%, 30.75% and 39.68%, respectively). Groups treated with RBO (0.2 and 0.4 mL/rat) for two weeks post-induction of liver fibrosis showed a significant (p≤0.05) reduction of liver TGF-β1 (5902.0±440.72 and 4905.7±305.98 pg/ml), FAK (11935.1±1079.75 and 11204.8±734.83 pg/mL) and α-SMA (616.4±34.81 and 11204.8±734.83 pg/mL) as compared to control positive group and showing % of reduction in TGF-β (27.5% and 39.74%), FAK (20.55% and 25.41%) and α-SMA (24.04% and 24.86%). Groups treated with RBO (0.4 mL/rat) showed no considerable change with the group treated with silymarin in TGF-β1 and FAK (5351.6±240.28 and 11889.6±471.32 pg/mL, respectively) with % of reduction of TGF-β1 and FAK (34.26% and 20.85%, respectively). Whereas, the group treated with RBO (0.4 mL/rat) showed better results than that exerted by silymarin, which still showed significant elevation (647.2±34.93 ng/mL) compared to the negative control % reduction =20.24% as compared to control positive group.  **Histopathological investigation**  The histological structure of portal regions, central veins, and hepatic parenchymal cells were normal in control rats' livers, livers of rats given thioacetamide revealed significant fibroplasia, which began in the portal triads, accompanied by bile duct epithelial proliferation, vascular congestion, and mononuclear inflammatory cells infiltration, which showed peripheral extension toward the parenchyma as fibrous bands that resulted in marked parenchymal pseudo-lobulation. The hepatic cells within those pseudo-lobules showed vacuolar degeneration with eccentric nuclei, necrosis. Also, Apoptosis along with inflammatory infiltrates was obviously seen along the fibrous septa.  Regarding livers of various treated groups, variable degrees of retraction of fibrous proliferation were noticed, the best of which was detected in the RBO-treated group at the high dose (0.4 ml/rat). Livers of low dose RBO-treated (0.2 ml/rat) group revealed mild fibroplasia in the portal regions, along with bile duct epithelial proliferation and a few inflammatory cells infiltration. A moderate degree of hepatocellular degeneration, scattered necrosis, and apoptosis was seen. Livers of RBO-treated rats with a high dose (0.4 ml/rat) revealed scarce fibroplasia in the portal areas with minimal changes and good restoration of the hepatic cells.  Livers of Silymarin-treated rats exhibited moderate fibroplasia in the portal regions, with peripherally expanded incomplete septa, few inflammatory cells infiltration, and cholangiolar proliferation. A moderate degree of hepatocellular degenerative and necrotic alterations was noticed. The scoring of fibroplasia extension was evaluated using the Metavir scoring system (ranged from F0=no fibrosis to F4=cirrhosis) in all of the experimental groups.  **Immunohistochemical investigation**  Livers of the model fibrosis (TAA-induced) group showed marked expression of both p-Akt and PDGF-BB compared to the control group. While RBO-treated groups (0.2 and 0.4 mL/rat) two-week post liver fibrosis induction showed marked dose-related deceased immuno-expression of p-Akt and PDGF-BB, particularly in the high dose treated group compared to the high dose TAA group and silymarin treated group. Silymarin treated group showed decreased expression of both markers. The quantitative analysis of the positive brown color of p-Akt and PDGF-BB, expressed as staining score, demonstrated positive (p≤0.05) overexpression in the TAA-treated group compared to the other treatment groups.  **Effects of RBO against TAA-Induced NF-κB and COX-2 Activation**  In this study, expression levels of NF-кB and COX-2 inflammatory mediators in the liver were analyzed by qRT-PCR. The TAA-intoxicated group showed significant upsurges in the content of the mRNA of NF-кB (3.67-fold) as compared to the negative control value. Treatment of TAA-intoxicated rats with silymarin, RBO (0.4 mL/rat), and (0.2 mL/rat) significantly downregulated hepatic NF-кB expression (1.26, 1.61, and 2.07-fold) as compared to the TAA group. Similarly, TAA intoxication resulted in a substantial increase in hepatic COX-2 to 2.43-fold as compared to negative control values. Supplementation with silymarin, RBO (0.4 mL/rat), and (0.2 mL/rat) significantly restored COX-2 expression to 0.94, 1.74, and 1.92-fold. | **10- Results** |
| The objective of the current study is to investigate the effect of rice bran oil (RBO) on hepatic fibrosis as a characteristic response to persistent liver injuries. Rats were randomly allocated into five groups: the negative control group, thioacetamide (TAA) group (thioacetamide 100 mg/kg thrice weekly for two successive weeks, ip), RBO 0.2 and 0.4 groups (RBO 0.2mL and 0.4 mL/rat/day, po) and standard group (silymarin 100 mg/kg/day, po) for two weeks after TAA injection. Blood and liver tissue samples were collected for biochemical, molecular, and histological analyses. Liver functions, oxidative stress, inflammation, liver fibrosis markers were assessed. The obtained results showed that RBO reduced TAA-induced liver fibrosis and suppressed the extracellular matrix formation. Compared to the positive control group, RBO dramatically reduced blood levels of total bilirubin, AST and ALT. Furthermore, RBO reduced MDA and increased GSH contents in the liver. Simultaneously RBO downregulated the NF-κB signaling pathway, which in turn inhibited the expression of some inflammatory mediators, including Cox-2, IL-1β, and TNF-α. RBO attenuated liver fibrosis by suppressing the biological effects of TGF-β1, α-SMA, collagen I, hydroxyproline, CTGF, and focal adhesion kinase (FAK). RBO reduced liver fibrosis by inhibiting hepatic stellate cell activation, and modulating the interplay among the TGF-β1 and FAK signal transduction. The greater dosage of 0.4 mL/kg has a stronger impact. Hence, this investigation presents RBO as a promising anti-fibrotic agent in TAA model through inhibition of TGF-β1 /FAK/α-SMA.  Keywords: Liver fibrosis; Thioacetamide; Rice bran oil; Focal adhesion kinase; oxidative stress and inflammation. | **11- Abstract** |
| Liver fibrosis is known as a dynamic process to repetitive liver injury, which eventually leads to cirrhosis and organ failure [1]. It is a considerable health problem that is associated with significant morbidity and mortality worldwide. The principal causative factors of liver fibrosis in developing countries are hepatitis C virus and/or parasitic infections while in the developed countries, the frequent causes are hepatitis B virus and excessive alcohol consumption [2]. Numerous stimuli are known to cause chronic liver inflammation and hepatic fibrosis, including but not limited to autoimmune disorders, metabolic disorders, drug or toxins-induced diseases, chronic cholestatic diseases, and genetic diseases [3]. Despite various etiologies, fibrosis represents a hallmark of all chronic liver diseases; if left, it progresses to cirrhosis, hepatocellular carcinoma, and eventually death [4]. Progressive liver fibrosis represents the major risk factor for developing liver-related complications and mortality [5].  Hepatic stellate cells (HSCs) are cells that exists in the space of Disse among hepatocytes and sinusoidal endothelial cells in the liver. One of its distinguishing characteristics is that they retain vitamin A lipids in their cytosol [6]. Upon repeated liver injury, quiescent HSCs become activated and differentiated into myofibroblast‐like cells; they lose their stored vitamin A and lipids; express α-smooth muscle actin (α-SMA); and produce large amounts of extracellular matrix (ECM) proteins [7]. A variety of cellular and molecular mechanisms implicated in HSC activation and consequent fibrogenesis, including cytokines and reactive oxygen species, thus offers probable therapeutic targets [2,8]. Among the multiple signaling pathways that participate in the fibrogenic process is the focal adhesion kinase (FAK) signaling pathway.  Focal adhesion kinase is a cytoplasmic tyrosine kinase that plays a pivotal role in liver fibrosis via activation and differentiation of HSC, stimulation of myofibroblast proliferation, and resistance to apoptosis. Additionally, inhibition of FAK signaling by drugs may be a potential target for preventing liver fibrosis [9]. FAK activation is vital for expression of α-SMA and pro-fibrotic collagens from hepatic stellate cells -[10]. In the fibrotic liver, the FAK mRNA levels are higher than in the healthy liver [11]. FAK is a potential mediator of fibrosis via fibroblast attachment to the extracellular matrix (ECM); and according to previous studies, it downregulates signaling that is implicated in two main fibrosis mechanisms. Phosphatidylinositol 3-kinase/protein kinase B (PI3K/AKT) signaling pathway is known as a mammalian target of rapamycin/S6 kinase (mTOR/S6K) complex. At the same time, the extracellular signal is controlled by ERK1/2, which is one of mitogen-activated protein kinase (MAPK) signal transduction. These pathways can result in α-SMA-positive myofibroblast diversity as well as collagen formation of various types, indicating the progression of liver fibrosis [12]. As a result, pharmacological suppression of FAK might be a viable treatment option for liver fibrosis. In this case, natural bioactive components from plant materials, particularly plant products, may be helpful. Meanwhile, RBO, which is derived from such plant sources, is a promising candidate [13].  Rice bran oil is becoming more widely used around the globe. It is a rice processing industry by-product that is removed from the white rice during the curing process [14]. Oryzanols, tocotrienols, tricin, phytosterols, policosanols, squalene, tocopherols, and ferulic acid are only some of the bioactive components that are found in RBO [15,16]. Various health advantages have been claimed for RBO, particularly cholesterol-lowering [17], anti-inflammatory [18], and antioxidant activities [19]. Additionally, RBO is edible oil that is available in the local market and is rich in many commercially and nutritionally important phytochemicals such as oryzanol, lecithin, and tocotrienols. Many of these phytochemicals are removed as waste by-products of RBO during the refining process. One of such components is γ-oryzanol which is a mixture of ferulic acid esters of sterol and triterpene alcohols [20]. RBO contains oryzanol at a level of 1 to 2%, where it acts as a natural antioxidant that can reduce blood cholesterol levels and lowering the risk of coronary heart disease [21]. The chemo-preventive activity of rice bran- derived components has been related to bioactive phytochemicals such as ferulic acid, tocotrienols/tocopherols, tricin, β-sitosterol, γ-oryzanol, and phytic acid. The underlying mechanisms may be mediated through their ability to inhibit cell proliferation, induce apoptosis, and to alter cell cycle progression in cancer cells. Moreover, rice bran bioactive components could protect against tissue damage through the radical scavenging activity and via blocking the chronic inflammatory responses [22].  RBO is a balanced source of saturated fatty acids (SFA: 20% palmitic acid), monounsaturated (MUFA: 42% oleic acid), and polyunsaturated fatty acid (PUFA: 32% linoleic acid) with an average ratio of 0.6:1.1:1.0, respectively. Moreover, it is of better oxidative stability than other cooking oils, with a high smoke point of 232 ºC and an ignition point of 350 ºC, making it suitable for the high-temperature cooking process [23]  Interestingly, rice bran and its oil are rich in tricin, a natural flavonoid with anti-inflammatory properties that shows a variety of biological actions by inhibiting NF-κB signaling and therefore reducing the release of pro-inflammatory cytokines [24,25]. Moreover, tricin exhibited anticancer effects via suppression of FAK protein levels and its downstream signals [26]. | **12- Background** |
| To date, no data is available to link the regulation of TGF-β1 /FAK/α-SMA pathway to the anti-fibrotic value of rice bran oil against TAA-induced liver fibrosis. Therefore, the current study aims at scouting the potential anti-fibrotic effects of RBO against TAA-induced liver fibrosis in rats. The study also investigated the involvement of the TGF-β1/FAK/α-SMA pathway to TAA-induced liver fibrosis and whether the selected oil could abate this dysregulation and suppress inflammatory mediators and oxidative stress. | **13- Objectives** |
| The animals were treated according to the national and international ethics guidelines. All experimental methods were carried out in compliance with the ethical criteria authorized by the “NRC's Committee on Animal Care and Use's ethics committees” (approval number: MREC-19-214). | **14- Ethical statement** |
| Rats were kept in plastic cages in breeding unit at National Research Centre at room temperature (25 °C) with a 12 h light and 12 h dark cycle. | **15- Housing and husbandry** |
| 1. Animals that are subject to procedures that cause no pain or distress, or only momentary or slight pain or distress and do not require the use of pain‐relieving drugs 2. Animals will be monitored throughout the experiment for unexpected adverse effects 3. Study will stop in case of unusual pain, wound or death of the animals. | **16- Animal care and monitoring** |
| Thioacetamide (TAA) is an organosulfur compound that has been widely used to induce experimental liver injury and liver fibrosis. TAA gets metabolized into thioacetamide-S-oxide and acetamide immediately after administration to rats [2]. Thioacetamide-S-oxide, the metabolic intermediate of TAA, binds to certain macromolecules in the cell that are responsible for the change in cell permeability and interruption of calcium stores, and it also inhibits mitochondrial activity, eventually leading to cellular damage and hepatocyte necrosis [37,38]. TAA is well-known hepatotoxin produces hardly reversible fibrosis in rodents similar to that of human, for that, it is an ideal model to test potential anti-fibrotic drugs [39]. Previous studies showed that TAA induced liver fibrosis through oxidative stress, evidenced by the elevation of MDA and suppression of GSH and SOD. TAA also upsurges the expression of α-SMA, TGF-β1, and PI3K/Akt pathway [40].  Rice bran oil (RBO) is rich in many bioactive phytochemicals such as γ-oryzanols, tocopherols, tocotrienols, carotenoids, phytosterols, squalene, policosanols, ferulic acid, and unsaturated fatty acids [13,41]. Furthermore, it could exhibit hypocholesterolemic, hypolipidemic, anti-inflammatory, cytotoxic, and antioxidant effects. As well, RBO contains nearly 38% oleic acid, 34% linoleic acid, and 18.6% palmitic acid [42].  Two weeks of TAA injection (100 mg/kg) three times weekly resulted in a significant increase in the activity of liver marker enzymes (ALT, AST) along with suppression in albumin and total proteins levels. Elevation of the activities of liver transaminases following TAA administration denotes liver cellular leakage and diminution in the structural and functional integrity of liver cells and thus designated as an index of damage of the liver parenchyma cells [39,43].  Our results implied that RBO administration significantly decreased serum transaminases, which is consistent with the previous findings of Rana et al. ‎[44], where RBO significantly decreased the activities of the enzymes of liver function test (AST, ALT, ALP) in N-nitrosodiethylamine-induced oxidative stress in rats.  Serum levels of albumin and total proteins reflect the functional status of hepatocytes [45]. TAA poisoning causes ubiquitin-associated protein degradation, which might be one of the main reasons for a drop in total protein levels in the blood. TAA treatment resulted in substantial reductions in serum total protein, albumin levels, and the A/G ratio in the current investigation. Hypoproteinemia might also be caused by an inflammatory response or a disruption in protein production in the fibrotic liver. Furthermore, free radicals and toxic metabolites generated by TAA are responsible for cellular death and the liver's incapability to execute its metabolic and excretory functions [1].  Compared to the positive control group, the RBO adjusted A/G ratio considerably boosted total protein and albumin levels. These results are consistent with those obtained by Abd Allah et al. [46], who cited that RBO normalized serum albumin, globulins, total proteins, and A/G ratio, owing to its hepatoprotective activity.  TAA-induced liver fibrosis is exacerbated by oxidative damage, which has been identified as a key molecular cause of TAA-induced hepatotoxicity. Oxidative agents and lipid peroxidation products aid the production of profibrogenic growth factors, cytokines, and prostaglandins. Additionally, reactive oxygen species (ROS), which include the epithelium, activated inflammatory cells, and/or microvascular endothelium, contribute to liver injury [47]. GSH is a non-enzymatic antioxidant found in hepatocytes that shield the membrane protein thiols of liver cells from the ROS damaging effects such as hydrogen peroxide and superoxide radicals. When GSH is reduced due to oxidation, it is transformed to glutathione disulfide, which causes lipid peroxidation [45].  In this work, rats treated with TAA had substantially lower hepatic GSH content, restored by RBO or silymarin treatment. Furthermore, as compared to the negative control group, TAA injection enhanced liver MDA content. When compared to the TAA-treated group, MDA content was substantially lower in the RBO or silymarin-treated groups. These findings are consistent with Al-Okbi et al. [13]; they claimed that RBO might successfully protect against liver inflammation and oxidation caused by a high-fat diet, as seen by MDA and (Tumor necrosis factor-α) TNF-α reductions.  In the current study, hepatic TNF-α, IL-1β, NF-κB, Collagen I, CTGF, and Hydroxyproline levels in the TAA group were significantly elevated, compared to the negative group.  Tumor necrosis factor-α is a multifunctional cytokine contributing to chronic progress of liver inflammation that accounts for liver fibrosis. TNF-α causes the activation of local HSCs into fibrogenic myofibroblasts during the inflammatory phase [1]. Moreover, IL-1β, a member of the interleukin-1 superfamily generated by the liver, leads to the activation of resident immune cells and the migration of other leukocytes to the injured liver, resulting in chronic inflammation, along with pro-inflammatory IL-6 and TNF-α.  Compared to the positive control group, treatment of RBO at both dosage levels (0.2 and 0.4 mL/rat) for two weeks after development of liver fibrosis made a considerable reduction in liver content of TNF-α, IL-1β, and NF-κB. Furthermore, tocotrienol, a component of RBO, has been shown to block the inflammatory transcription factor NF-kB, which is connected to inhibiting chronic inflammation, pro-fibrotic cytokines, apoptosis, and oxygen free radical-induced damage [48].  The pro-inflammatory cytokine regulator, NF-κB, is suggested to be a master contributor to liver inflammation and fibrogenesis processes through regulation of pro-inflammatory cytokines production and HSCs activation [49]. In normal conditions, NF-κB is sequestered in the cytoplasm by the inhibitory protein I-κB which inhibits NF-κB from nuclear translocation. Upon liver injury, I-κB undergoes phosphorylation and degradation; thus NF-κB is activated and translocated into the nucleus, where it induces the transcription of hundreds of target genes involved in the regulation of inflammatory and immune responses [50].  NF-κB pathway activation has been reported to be associated with enhanced liver fibrogenesis through the stimulation of pro-inflammatory mediators such as TNFα, IL-6, iNOS, COX-2, PGE2, and MMP-9 [51]. Our findings are in agreement with previously reported studies that TAA activated NF-κB. However, RBO treatment significantly modulated TAA-induced NF-κB upregulation.  Interestingly, the phytoconstituent γ-oryzanol from RBO, repressed NF-ĸB initiation and downregulated the inflammatory responses of the macrophage cell line [13,52].  COX-2 is one of NF-κB downstream inflammatory targets induced by various stimuli, including oxidative stress, inflammation, and carcinogens [53]. Previous studies indicated that COX2 activation with the subsequent PGs release favors the development of the necroinflammatory condition, HSCs proliferation, and angiogenesis that could contribute to hepatic fibrosis and carcinogenesis [54]. Thereby, the mechanism of some anti-fibrotic and antiproliferative agents such as celecoxib is mediated via inhibiting COX-2 in HSCs with a consequent reduction in PGs [54]. Our results showed that RBO treatment significantly decreased mRNA expression of COX-2 and TGF-β1, as well as reduced hepatic collagen deposition compared to TAA-treated rats. These findings suggest RBO as an anti-fibrotic agent of hepatic fibrosis.  Abnormal synthesis and accumulation of type I collagen in the extracellular matrix is considered the end product of fibrosis, produced by activated stellate or Ito cells in the damaged liver. Furthermore, hydroxyproline, a characteristic amino acid present in collagen, is the major component of the collagen triple helix. It reflects the total collagen content in the liver tissue. Hydroxyproline is used as a marker to determine the degree of fibrosis and assess the efficacy of new anti-fibrotic drugs [55].  Liver hydroxyproline content was markedly amplified in the TAA intoxicated group compared to the negative control group (p≤0.05). Similarly, collagen I was significantly upregulated in the livers of rats in the TAA administered group compared with the negative control group (p≤0.05).  RBO revealed a significant reduction of liver collagen I as compared to the fibrotic group.  Supporting our findings, Phetpornpaisan et al. ‎[56] mentioned that the ethanolic extract of rice bran is rich in bio-active elements including caffeic acid, cyanidin-3-glucoside, ferulic acid and p-coumaric acid, according to their study. Furthermore, rice bran extract demonstrated antioxidation, MMP-2 and MMP-9 inhibition, wound healing benefits as evidenced by increased fibroblast formation and collagen production, as well as the immunomodulatory effect.  Additionally, HSCs and hepatocytes produce “connective tissue growth factor (CTGF, also known as CCN2)”, which is highly expressed during liver fibrosis. CTGF, a powerful fibrotic activity, is a key regulator of TGF-β1 activator, increasing the cytokine's fibrogenic effects, especially in the hepatic cells and other places. Moreover, hepatocytes synthesize CTGF in the damaged liver; and TGF‐β1 stimulated hepatocytes appear to be the principal cellular source of CTGF in the liver [19]. Moreover, a positive correlation between gene expression of TGF-β1, CTGF, and α-SMA supports their involvement in the fibrogenic mechanism. TGF-β1 is the most potent inducer of CCN2; additionally, CCN2 is known to act downstream of the TGF-β1 signaling pathway. The promoter activity CCN2 is upregulated not only by TGF-β1 but also by PDGF, ethanol, and acetaldehyde [57].  Rats treated with RBO after induction of liver fibrosis showed a significant reduction of liver content of hydroxyproline, collagen I, and CTGF compared to the positive control group. RBO is rich in antioxidant phytoconstituents such as tocotrienol, tocopherols, and many others that could protect against hepatic injury. Balah et al. ‎[58] reported that vitamin E inhibited cyclosporin A (CsA)-induced TGF-β1/Smad signaling pathway and subsequent suppression of CTGF and TIMP-1 expression in rat liver.  In the fibrotic liver, TGF-β1 is secreted by both autocrine and paracrine cells. TGF-1 stimulates collagen, α-SMA, and other ECM proteins transcription and release [59]. Confirming this, TGF-β1 and α-SMA expression were substantially increased in TAA-intoxicated rats.  The cytoplasmic protein tyrosine kinase FAK is a non-receptor cytoplasmic protein tyrosine kinase. When cells connect to ECM proteins through integrin binding, FAK is activated. Integrins are the principal adhesion receptors that transfer signals among ECM pathways and their cytoplasmic domains across the cell plasma membrane. FAK is also activated in response to TGF-β1 activation and also other cytokines and growth factors [9,60]. Consistent with the findings of the current study, TGF-β1 activates HSCs to produce fibronectin, α-SMA, and CTGF, markers of HSC activation. Therefore, FAK activation in fibrotic liver tissue is associated with increased α-SMA and collagen production [60]. Similarly, active FAK increases invasion, as well as myofibroblast development and resistance to apoptosis, in chronic liver disorders, suggesting its contribution to liver fibrosis [9]. | **17- Interpretation/scientific implication** |
| Throughout the four-week of the current investigation, RBO displayed beneficial protective therapy against TAA-induced liver fibrosis. RBO amended oxidation-induced damage via dropping down of lipid peroxidation and elevating GSH. Furthermore, RBO substantially curtailed inflammation by regulation of TNF-α, IL-6, NF-κB, COX2, pAKT, PDGF which contributes to the downregulation of the TGF-β1 pathway. Most significantly, RBO inhibited the TGF-β1/FAK signaling pathway, which reduced HSC growth and division. Finally, RBO inhibited ECM deposition and fibrosis progression by suppressing fibrogenic factors expression as TGF-β1, α-SMA, collagen I, CTGF and PDGF. More research is warranted to understand the connection between RBO and the TGF-β1/FAK pathway in the prevention of liver fibrosis, as well as additional pathways that mediate RBO's anti-fibrotic activity. | **18- Generalisability/translation** |
| A research idea has been submitted through the research plan of the National Research Centre, and a detailed protocol has been submitted on the idea of ​​the project, purpose of conducting research experiments in addition to the material needed and methods of the work in the research project. Project No. 12060127 has been approved in addition to the approval of the Ethics Committee of the National Research Centre for experiments on Laboratory animals, and then work on the research project began. | **19- Protocol registration** |
| The authors have declared that no conflict of interests exists | **20- Declaration of interest** |
| 1. Ramadan A, Afifi N, Yassin NZ, Abdel-Rahman RF, Abd El-Rahman SS, Fayed HM. Mesalazine, an osteopontin inhibitor: The potential prophylactic and remedial roles in induced liver fibrosis in rats. Chem Biol Interact. 2018;289: 109–118. doi:10.1016/j.cbi.2018.05.002  2. Hessin A, Hegazy RR, Hassan AA, Yassin NZ, Kenawy SAB. Resveratrol prevents liver fibrosis via two possible pathways: Modulation of alpha fetoprotein transcriptional levels and normalization of protein kinase C responses. Indian J Pharmacol. 2017;49: 282–289. doi:10.4103/ijp.IJP_299_16  3. Tovar R, Flores-Beltrán RE, Favari L, Muriel P, Zarco N, Segovia J, et al. Naringenin prevents experimental liver fibrosis by blocking TGFβ-Smad3 and JNK-Smad3 pathways. World J Gastroenterol. 2017;23: 4354–4368. doi:10.3748/wjg.v23.i24.4354  4. Raad II, Chaftari AM, Torres HA, Ayoub EM, Narouz LI, Bartek J, et al. Challenge of hepatitis C in Egypt and hepatitis B in Mauritania. World Journal of Hepatology. Baishideng Publishing Group Inc; 2018. pp. 549–557. doi:10.4254/WJH.V10.I9.549  5. Sharkawy R El, Bayoumi A, Metwally M, Mangia A, Berg T, Romero-Gomez M, et al. A variant in the MICA gene is associated with liver fibrosis progression in chronic hepatitis C through TGF-β1 dependent mechanisms. Sci Rep. 2019;9: 1439. doi:10.1038/s41598-018-35736-2  6. Asahina K. Hepatic stellate cell progenitor cells. J Gastroenterol Hepatol. 2012;27: 80–84. doi:10.1111/j.1440-1746.2011.07001.x  7. Abd El‐Rahman SS, Fayed HM. Targeting AngII/AT1R signaling pathway by perindopril inhibits ongoing liver fibrosis in rat. J Tissue Eng Regen Med. 2019;13: 2131–2141. doi:10.1002/term.2940  8. Weiskirchen R, Tacke F. Liver Fibrosis: From Pathogenesis to Novel Therapies. Dig Dis. 2016;34: 410–422. doi:10.1159/000444556  9. Zhao X-K, Yu L, Cheng M-L, Che P, Lu Y-Y, Zhang Q, et al. Focal Adhesion Kinase Regulates Hepatic Stellate Cell Activation and Liver Fibrosis. Sci Rep. 2017;7: 4032. doi:10.1038/s41598-017-04317-0  10. Weng Y, Lieberthal TJ, Zhou VX, Lopez-Ichikawa M, Armas-Phan M, Bond TK, et al. Liver epithelial focal adhesion kinase modulates fibrogenesis and hedgehog signaling. JCI Insight. 2020;5. doi:10.1172/jci.insight.141217  11. Yuan Z, Zheng Q, Fan J, Ai K, Chen J, Huang X. Expression and prognostic significance of focal adhesion kinase in hepatocellular carcinoma. J Cancer Res Clin Oncol. 2010;136: 1489–1496. doi:10.1007/s00432-010-0806-y  12. Fan GP, Wang W, Zhao H, Cai L, Zhang P De, Yang ZH, et al. Pharmacological Inhibition of Focal Adhesion Kinase Attenuates Cardiac Fibrosis in Mice Cardiac Fibroblast and Post-Myocardial-Infarction Models. Cell Physiol Biochem. 2015;37: 515–526. doi:10.1159/000430373  13. Al-Okbi S, Mohamed D, Hamed T, Esmail R. Rice Bran Oil and Pumpkin Seed Oil Alleviate Oxidative Injury and Fatty Liver in Rats Fed High Fructose Diet. Polish J Food Nutr Sci. 2014;64: 127–133. doi:10.2478/pjfns-2013-0002  14. Zavoshy R, Noroozi M, Jahanihashemi H. Effect of low calorie diet with rice bran oil on cardiovascular risk factors in hyperlipidemic patients. J Res Med Sci Off J Isfahan Univ Med Sci. 2012;17: 626–631.  15. Khatoon S, Gopalakrishna AG. Fat-soluble nutraceuticals and fatty acid composition of selected Indian rice varieties. J Am Oil Chem Soc. 2004;81: 939–943. doi:10.1007/s11746-004-1005-5  16. Ardiansyah, Shirakawa H, Koseki T, Ohinata K, Hashizume K, Komai M. Rice Bran Fractions Improve Blood Pressure, Lipid Profile, and Glucose Metabolism in Stroke-Prone Spontaneously Hypertensive Rats. J Agric Food Chem. 2006;54: 1914–1920. doi:10.1021/jf052561l  17. Chen CW, Cheng HH. A rice bran oil diet increases LDL-receptor and HMG-CoA reductase mRNA expressions and insulin sensitivity in rats with streptozotocin/nicotinamide- induced type 2 diabetes. J Nutr. 2006;136: 1472–1476. doi:10.1093/jn/136.6.1472  18. Xu Z, Hua N, Godber JS. Antioxidant activity of tocopherols, tocotrienols, and gamma-oryzanol components from rice bran against cholesterol oxidation accelerated by 2,2’-azobis(2-methylpropionamidine) dihydrochloride. J Agric Food Chem. 2001;49: 2077–2081. doi:10.1021/jf0012852  19. Akihisa T, Yasukawa K, Yamaura M, Ukiya M, Kimura Y, Shimizu N, et al. Triterpene alcohol and sterol ferulates from rice bran and their anti- inflammatory effects. J Agric Food Chem. 2000;48: 2313–2319. doi:10.1021/jf000135o  20. Bumrungpert A, Chongsuwat R, Phosat C, Butacnum A. Rice Bran Oil Containing Gamma-Oryzanol Improves Lipid Profiles and Antioxidant Status in Hyperlipidemic Subjects: A Randomized Double-Blind Controlled Trial. J Altern Complement Med. 2019;25: 353–358. doi:10.1089/acm.2018.0212  21. Scavariello EM, Arellano DB. [Gamma-oryzanol: an important component in rice brain oil]. Arch Latinoam Nutr. 1998;48: 7–12.  22. Henderson AJ, Ollila CA, Kumar A, Borresen EC, Raina K, Agarwal R, et al. Chemo-preventive properties of dietary rice bran: Current status and future prospects. Advances in Nutrition. 2012. pp. 643–653. doi:10.3945/an.112.002303  23. Punia, Sneh, Manoj Kumar, Anil Kumar Siroha and SSP. Rice Bran Oil: Emerging Trends In Extraction, Health Benefit, and Its Industrial Application. Rice Sci. 2021;28: 217–232. doi:10.1016/j.rsci.2021.04.002  24. Seki N, Toh U, Kawaguchi K, Ninomiya M, Koketsu M, Watanabe K, et al. Tricin inhibits proliferation of human hepatic stellate cells in vitro by blocking tyrosine phosphorylation of PDGF receptor and its signaling pathways. J Cell Biochem. 2012;113: 2346–2355. doi:10.1002/jcb.24107  25. Shalini V, Bhaskar S, Kumar KS, Mohanlal S, Jayalekshmy A, Helen A. Molecular mechanisms of anti-inflammatory action of the flavonoid, tricin from Njavara rice (Oryza sativa L.) in human peripheral blood mononuclear cells: possible role in the inflammatory signaling. Int Immunopharmacol. 2012;14: 32–38. doi:10.1016/j.intimp.2012.06.005  26. Chung DJ, Wang CJ, Yeh CW, Tseng TH. Inhibition of the Proliferation and Invasion of C6 Glioma Cells by Tricin via the Upregulation of Focal-Adhesion-Kinase-Targeting MicroRNA-7. J Agric Food Chem. 2018;66: 6708–6716. doi:10.1021/acs.jafc.8b00604  27. Wallace MC, Hamesch K, Lunova M, Kim Y, Weiskirchen R, Strnad P, et al. Standard operating procedures in experimental liver research: thioacetamide model in mice and rats. Lab Anim. 2015;49: 21–9. doi:10.1177/0023677215573040  28. Mehdi BJ, Tabassum S, Haider S, Perveen T, Nawaz A, Haleem DJ. Nootropic and anti-stress effects of rice bran oil in male rats. J Food Sci Technol. 2015;52: 4544. doi:10.1007/S13197-014-1489-1  29. Samad N. Rice bran oil prevents neuroleptic-induced extrapyramidal symptoms in rats: Possible antioxidant mechanisms. J Food Drug Anal. 2015;23: 370–375. doi:10.1016/J.JFDA.2014.10.012  30. Liping W, Li Z, Kelan G, Xiaoping H, Ying X, Yikun S, et al. Comparative effect on liver fibrosis of bicyclol and silymarin. African J Pharm Pharmacol. 2020;14: 77–86. doi:10.5897/ajpp2019.5085  31. Ellman GL. Tissue sulfhydryl groups. Arch Biochem Biophys. 1959;82: 70–77. doi:10.1016/0003-9861(59)90090-6  32. Ruiz-Larrea MB, Leal AM, Liza M, Lacort M, de Groot H. Antioxidant effects of estradiol and 2-hydroxyestradiol on iron-induced lipid peroxidation of rat liver microsomes. Steroids. 1994;59: 383–388. doi:10.1016/0039-128x(94)90006-x  33. Bancroft J d., Gamble M. Bancroft’s Theory and Practice of Histological Techniques. Bancroft’s Theory and Practice of Histological Techniques. Elsevier; 2019. doi:10.1016/C2015-0-00143-5  34. Ali AM, El-Tawil OS, Al-Mokaddem AK, Abd El-Rahman SS. Promoted inhibition of TLR4/miR-155/ NFkB p65 signaling by cannabinoid receptor 2 agonist (AM1241), aborts inflammation and progress of hepatic fibrosis induced by thioacetamide. Chem Biol Interact. 2021;336: 109398. doi:10.1016/j.cbi.2021.109398  35. Mohamed Ali A, Samir El-Tawil O, Samir Abd El-Rahman S. Inhibited TLR-4/NF- κB Pathway Mediated by Cannabinoid Receptor 2 Activation Curbs Ongoing Liver Fibrosis in Bile Duct Ligated Rats. Adv Anim Vet Sci. 2020;9: 253–264. doi:10.17582/journal.aavs/2021/9.2.253.264  36. El Badawy SA, Ogaly HA, Abd-Elsalam RM, Azouz AA. Benzyl isothiocyanates modulate inflammation, oxidative stress, and apoptosis via Nrf2/HO-1 and NF-κB signaling pathways on indomethacin-induced gastric injury in rats. Food Funct. 2021;12: 6001–6013. doi:10.1039/d1fo00645b  37. Liu Y, Meyer C, Xu C, Weng H, Hellerbrand C, ten Dijke P, et al. Animal models of chronic liver diseases. American Journal of Physiology - Gastrointestinal and Liver Physiology. Am J Physiol Gastrointest Liver Physiol; 2013. doi:10.1152/ajpgi.00199.2012  38. Zargar S, Wani TA, Alamro AA, Ganaie MA. Amelioration of thioacetamide-induced liver toxicity in Wistar rats by rutin. Int J Immunopathol Pharmacol. 2017;30: 207–214. doi:10.1177/0394632017714175  39. Ra SH, Shin RH, Ri HC, Ri JH, Ri HC, Ri AJ. Effect of lesimarin against thioacetamide-induced liver cirrhosis in rat. Brazilian J Pharm Sci. 2019;55: 17821. doi:10.1590/s2175-97902019000217821  40. Mi XJ, Hou JG, Jiang S, Liu Z, Tang S, Liu XX, et al. Maltol Mitigates Thioacetamide-induced Liver Fibrosis through TGF-β1-mediated Activation of PI3K/Akt Signaling Pathway. J Agric Food Chem. 2019;67: 1392–1401. doi:10.1021/acs.jafc.8b05943  41. Al-Okbi SY, Mohamed DA, Hamed TE, Al-Siedy ESK. Rice bran as source of nutraceuticals for management of cardiovascular diseases, cardio-renal syndrome and hepatic cancer. J Herbmed Pharmacol. 2020;9: 68–74. doi:10.15171/jhp.2020.10  42. Rukmini C, Raghuram TC. Nutritional and biochemical aspects of the hypolipidemic action of rice bran oil: a review. J Am Coll Nutr. 1991;10: 593–601. doi:10.1080/07315724.1991.10718181  43. El-Latif El-Ghazaly MA, Rashed ER, Shafey GM, Zaki HF, Attia AS. Amelioration of thioacetamide-induced hepatic encephalopathy in rats by low-dose gamma irradiation. Environ Sci Pollut Res. 2020;27: 334–343. doi:10.1007/s11356-019-06934-w  44. Rana P, Vadhera S, Soni G. In vivo antioxidant potential of rice bran oil (RBO) in albino rats. Indian J Physiol Pharmacol. 2004;48: 428–436.  45. Eldesoky AH, Abdel-Rahman RF, Ahmed OK, Soliman GA, Saeedan AS, Elzorba HY, et al. Antioxidant and hepatoprotective potential of Plantago major growing in Egypt and its major phenylethanoid glycoside, acteoside. J Food Biochem. 2018;42: e12567. doi:10.1111/jfbc.12567  46. Abd Allah H, Abd El-wahab B, Ramadan K, Ali S. GINGER ETHANOLIC EXTRACT, GINGER OIL OR RICE BRAN OIL INDUCED HEPATOPROTECTIVE EFFECT AGAINST FATTY LIVER IN RATS. Arab Univ J Agric Sci. 2018;26: 1135–1150. doi:10.21608/ajs.2018.28366  47. Yang HY, Kim KS, Lee YH, Park JH, Kim J-H, Lee S-Y, et al. Dendropanax morbifera Ameliorates Thioacetamide-Induced Hepatic Fibrosis via TGF-β1/Smads Pathways. Int J Biol Sci. 2019;15: 800–811. doi:10.7150/ijbs.30356  48. Haghighat N, Vafa M, Eghtesadi S, Heidari I, Hosseini A, Rostami A. The effects of tocotrienols added to canola oil on microalbuminuria, inflammation, and nitrosative stress in patients with type 2 diabetes: A randomized, double-blind, placebo-controlled trial. Int J Prev Med. 2014;5: 617–623. doi:10.1016/j.jacc.2013.08.648  49. Luedde T, Schwabe RF. NF-κB in the liver-linking injury, fibrosis and hepatocellular carcinoma. Nature Reviews Gastroenterology and Hepatology. Nat Rev Gastroenterol Hepatol; 2011. pp. 108–118. doi:10.1038/nrgastro.2010.213  50. Hoffmann A, Natoli G, Ghosh G. Transcriptional regulation via the NF-κB signaling module. Oncogene. 2006;25: 6706–6716. doi:10.1038/sj.onc.1209933  51. Xiao J, Ho CT, Liong EC, Nanji AA, Leung TM, Lau TYH, et al. Epigallocatechin gallate attenuates fibrosis, oxidative stress, and inflammation in non-alcoholic fatty liver disease rat model through TGF/SMAD, PI3 K/Akt/FoxO1, and NF-kappa B pathways. Eur J Nutr. 2014;53: 187–199. doi:10.1007/s00394-013-0516-8  52. Nagasaka R, Chotimarkorn C, Shafiqul IM, Hori M, Ozaki H, Ushio H. Anti-inflammatory effects of hydroxycinnamic acid derivatives. Biochem Biophys Res Commun. 2007;358: 615–619. doi:10.1016/j.bbrc.2007.04.178  53. Sung YK, Hwang SY, Kim JO, Bae HI, Kim J-C, Kim MK. The correlation between cyclooxygenase-2 expression and hepatocellular carcinogenesis. Mol Cells. 2004;17: 35–38.  54. Ftahy MM, Latif NSA, Alalkamy EF, El-Batrawi FA, Galal AH, Khatab HM. Anti-fibrotic potential of a selective COX-2 inhibitor (celecoxib) on liver fibrosis in rats. Comp Clin Path. 2013;22: 425–430. doi:10.1007/s00580-012-1427-4  55. Koilan S, Hamilton D, Baburyan N, Padala MK, Weber KT, Guntaka R V. Prevention of liver fibrosis by triple helix-forming oligodeoxyribonucleotides targeted to the promoter region of type i collagen gene. Oligonucleotides. 2010;20: 231–237. doi:10.1089/oli.2010.0244  56. Phetpornpaisan P, Tippayawat P, Jay M, Sutthanut K. A local Thai cultivar glutinous black rice bran: A source of functional compounds in immunomodulation, cell viability and collagen synthesis, and matrix metalloproteinase-2 and -9 inhibition. J Funct Foods. 2014;7: 650–661. doi:10.1016/j.jff.2013.12.020  57. Shimizu K. Mechanisms of pancreatic fibrosis and applications to the treatment of chronic pancreatitis. Journal of Gastroenterology. Japan; 2008. pp. 823–832. doi:10.1007/s00535-008-2249-7  58. Balah A, Ezzat O, Akool E-S. Vitamin E inhibits cyclosporin A-induced CTGF and TIMP-1 expression by repressing ROS-mediated activation of TGF-β/Smad signaling pathway in rat liver. Int Immunopharmacol. 2018;65: 493–502. doi:10.1016/j.intimp.2018.09.033  59. KA E-M, HI K, A E-K, NM E, LA E. Naringin attenuates thioacetamide-induced liver fibrosis in rats through modulation of the PI3K/Akt pathway. Life Sci. 2017;187: 50–57. doi:10.1016/J.LFS.2017.08.019  60. Chen Y, Li Q, Tu K, Wang Y, Wang X, Liu D, et al. Focal Adhesion Kinase Promotes Hepatic Stellate Cell Activation by Regulating Plasma Membrane Localization of TGFβ Receptor 2. Hepatol Commun. 2020;4: 268–283. doi:10.1002/hep4.1452 | **References** |
