# Supplementary figures and images for: The involvement of TGF-β1 /FAK/α-SMA pathway in the antifibrotic impact of rice bran oil on thioacetamide-induced liver fibrosis in rats
Source: PLoS One. 2021 Dec 29;16(12):e0260130. doi: 10.1371/journal.pone.0260130 (PMC8716044; doi:10.1371/journal.pone.0260130)

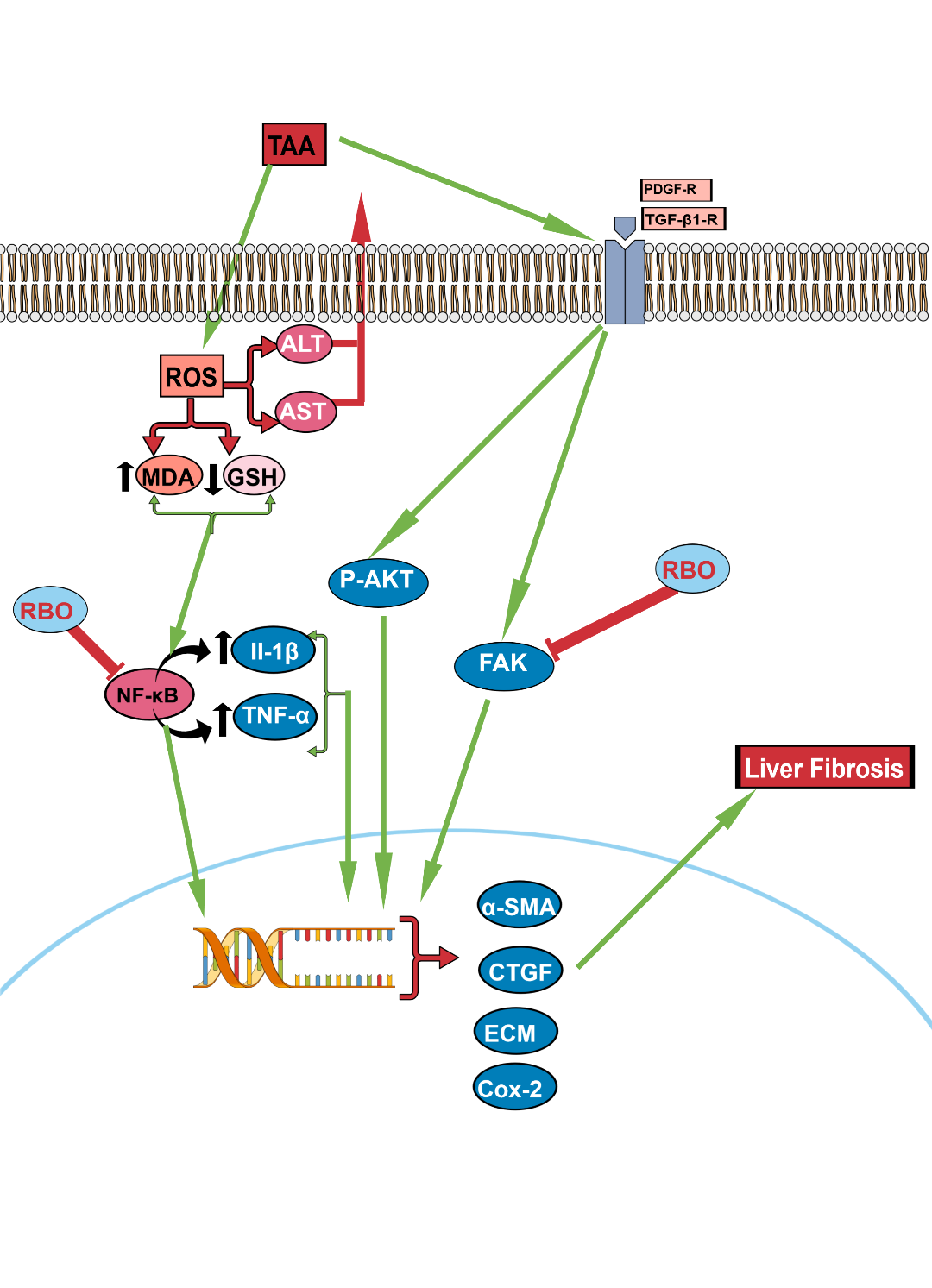

Supplement: S1 Graphical abstract — (PNG) [file pone.0260130.s003.png]
